# Autophagy in Myf5+ progenitors regulates energy and glucose homeostasis through control of brown fat and skeletal muscle development

**DOI:** 10.1038/embor.2013.111

**Published:** 2013-08-02

**Authors:** Nuria Martinez-Lopez, Diana Athonvarangkul, Srabani Sahu, Luisa Coletto, Haihong Zong, Claire C Bastie, Jeffrey E Pessin, Gary J Schwartz, Rajat Singh

**Affiliations:** 1 Department of Medicine, Albert Einstein College of Medicine, Bronx, New York 10461, USA; 2 Department of Molecular Pharmacology, Albert Einstein College of Medicine, Bronx, New York 10461, USA; 3 Venetian Institute of Molecular Medicine, 35129 Padova, Italy; 4 Diabetes Research Center, Albert Einstein College of Medicine, Bronx, New York 10461, USA; 5 Division of Metabolic and Vascular Health, Warwick Medical School, Coventry CV4 7AL, UK; 6 Department of Neuroscience, Albert Einstein College of Medicine, Bronx, New York 10461, USA; 7 Institute for Aging Studies, Albert Einstein College of Medicine, Bronx, New York 10461, USA

**Keywords:** Autophagy, Myf5+ progenitors, brown fat

## Abstract

Macroautophagy (MA) regulates cellular quality control and energy balance. For example, loss of MA in aP2-positive adipocytes converts white adipose tissue (WAT) into brown adipose tissue (BAT)-like, enhancing BAT function and thereby insulin sensitivity. However, whether MA regulates early BAT development is unknown. We report that deleting *Atg7* in myogenic Myf5+ progenitors inhibits MA in Myf5-cell-derived BAT and muscle. Knock out (KO) mice have defective BAT differentiation and function. Surprisingly, their body temperature is higher due to WAT lipolysis-driven increases in fatty acid oxidation in ‘Beige’ cells in inguinal WAT, BAT and muscle. KO mice also present impaired muscle differentiation, reduced muscle mass and glucose intolerance. Our studies show that ATG7 in Myf5+ progenitors is required to maintain energy and glucose homeostasis through effects on BAT and muscle development. Decreased MA in myogenic progenitors with age and/or overnutrition might contribute to the metabolic defects and sarcopenia observed in these conditions.

## Introduction

The metabolic syndrome is a major health issue affecting ∼25% of the US population [1]. While disturbances in energy balance contribute to the metabolic syndrome, the mechanisms leading to energy imbalance are unclear. Adipose tissues and skeletal muscle (SKM) have pivotal roles in regulating energy and glucose homeostasis [2]. Excess energy is stored as lipid in white adipose tissue (WAT), whereas brown adipose tissue (BAT) expends energy by generating heat [3]. SKM [2] and BAT [4] maintain glucose homeostasis via glucose uptake in response to insulin, and intriguingly, both tissues originate from myogenic factor 5-positive (Myf5+) progenitors [3]. It is thus conceivable that factors affecting Myf5+ progenitors will dysregulate energy balance through effects on BAT and SKM differentiation.

Macroautophagy (MA) entails formation of LC3-II-positive autophagosomes that sequester and target cytoplasmic cargo for lysosomal degradation [5]. In addition to quality control, MA regulates lipid metabolism by degrading lipid droplets (LD) via lipophagy [6]. Overnutrition and aging decrease MA in liver [6] and hypothalamic neurons [7], respectively, suggesting that metabolic defects in these conditions occur, in part, from reduced MA. MA also controls energy balance by regulating WAT differentiation [8]. Loss of a key MA gene, *Atg7*, in aP2+ adipocytes decreases WAT differentiation [8], and remarkably, *Atg7*^*−/−*^ WAT acquires BAT-like features [8]. As MA modulates WAT development, we asked whether MA in Myf5+ progenitors controls BAT development. Here we show that mice lacking *Atg7* in Myf5+ progenitors (Knock out, KO) show loss of MA in Myf5-derived tissues, BAT and SKM. Loss of *Atg7* disrupts BAT differentiation, and surprisingly, promotes ‘Beige’ (brown adipocyte-like) cell [9] development in inguinal (ing) WAT that contributes to increased energy expenditure and raised body temperature. KO mice show reduced SKM differentiation and mass and are glucose intolerant, thus revealing a key role for MA in Myf5+ progenitors in regulating energy and glucose homeostasis through effects on BAT and SKM development.

## Results and discussion

### Loss of *Atg7* in Myf5+ cells disrupts MA in BAT/SKM

**Figure 1:**
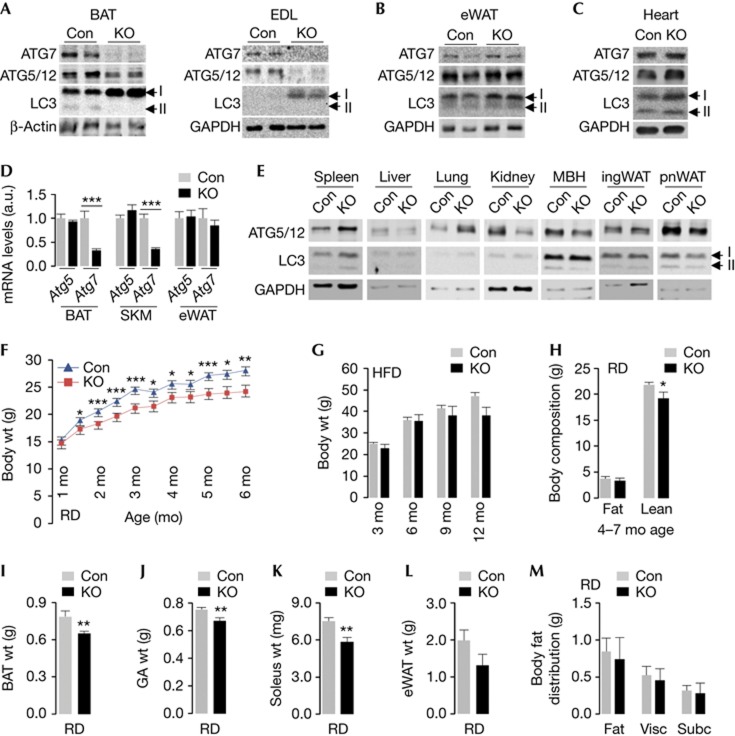
Deleting *Atg7* in Myf5+ progenitors disrupts macroautophagy (MA) in brown adipose tissue (BAT) and skeletal muscle (SKM). (**A**–**C**) Immunoblots for indicated proteins in BAT, extensor digitorum longus (EDL), epididymal white adipose tissue (eWAT) and heart from 10- to 12-month (mo)-old control (Con) and knock out (KO) mice. Arrows depict LC3-I and II. (**D**) ATG5 and ATG7 mRNA levels in indicated tissues (*n*=4), and (**E**) immunoblots for indicated proteins in spleen, liver, lung, kidney, mediobasal hypothalamus (MBH), inguinal white adipose tissue (ingWAT) and perinephric fat (pnWAT) from 10- to 12-mo-old Con and KO mice. (**F**) Body weights (wt) of chow diet (RD)-fed (*n*=6–29), and (**G**) high-fat diet (HFD)-fed Con and KO mice at indicated ages (*n*=4–17). (**H**) Total body fat and lean mass of 4–7 mo RD-fed Con and KO mice (*n*=8–12). (**I**) BAT wt (*n*=4–7), (**J**) gastrocnemius (GA) wt (*n*=5–7), (**K**) soleus wt (*n*=4–7), (**L**) eWAT wt (*n*=5–7) and (**M**) visceral (Visc) and subcutaneous (Subc) body fat distribution in 10-mo-old Con and KO mice (*n*=4). Values are mean±s.e., *P<0.05, **P<0.01, ***P<0.001.

To determine the effect of loss of MA during BAT development, we knocked out *Atg7* in Myf5+ progenitors by crossing *Atg7*^*Flox/Flox*^ [10] with Myf5-Cre mice [11]. KO mice displayed absence of ATG7, decreased pre-autophagosome-associated ATG5-ATG12 levels, LC3-I accumulation and loss of autophagosome-bound LC3-II in BAT and SKM (EDL, extensor digitorum longus; Fig 1A) without modifying those in epididymal (e) WAT or heart (Fig 1B,C). *Atg7* deletion in BAT and SKM was verified by qPCR analyses for diminished *Atg7* expression (Fig 1D), while those in eWAT (Fig 1D) or heart (supplementary Fig S1A online) remained unaffected. *Atg5* expression was comparable in tissues from control (Con) and KO mice (Fig 1D). Moreover, ATG5-ATG12 and LC3-II levels remained equivalent in spleen, liver, lung, kidney, mediobasal hypothalamus (MBH) and perinephric fat from Con and KO mice (Fig 1E). As small subsets of progenitors in ingWAT and eWAT express *myf5* [12], we failed to detect *Atg7* deletion in WAT from KO mice (Fig 1B). In fact, compensatory increases in ATG7 levels were detected in eWAT from KO mice (supplementary Fig S1B online), although increases in ATG7 did not enhance MA flux (not shown). Despite increased *Atg7* expression in ingWAT (supplementary Fig S1C online), ATG7 levels remained comparable in ingWAT from Con and KO mice (supplementary Fig S1B online).

The loss of MA in Myf5+ progenitors did not increase mortality or promote skeletal or neurological deficits indicated by absence of the hind-limb clasping reflex, although a mild resting tremor was observed in a subset of KO mice. KO mice on chow diet (RD) displayed decreased body weights as early as 6 weeks of age (Fig 1F) that associated with reduced (∼5%) nose-rump length. Both male (Fig 1F) and female KO mice (supplementary Fig S1D online) maintained lower body weights, although high-fat diet (HFD)-fed male Con and KO mice acquired comparable weights (Fig 1G). Decreased body weights in KO mice were largely from reduced lean mass as determined by quantitative NMR (qNMR; Fig 1H), although analyses of organ weights revealed decreased BAT (Fig 1I) and SKM weights (Figs 1J,K), and a trend towards smaller eWAT pads (Fig 1L). HFD-fed KO mice also displayed reduced lean mass when compared with Con (supplementary Fig S1E online). Furthermore, RD- (Fig 1M) or HFD-fed mice (supplementary Fig S1E online) did not redistribute fat between their visceral and subcutaneous depots.

### KO mice display impaired BAT differentiation

**Figure 2:**
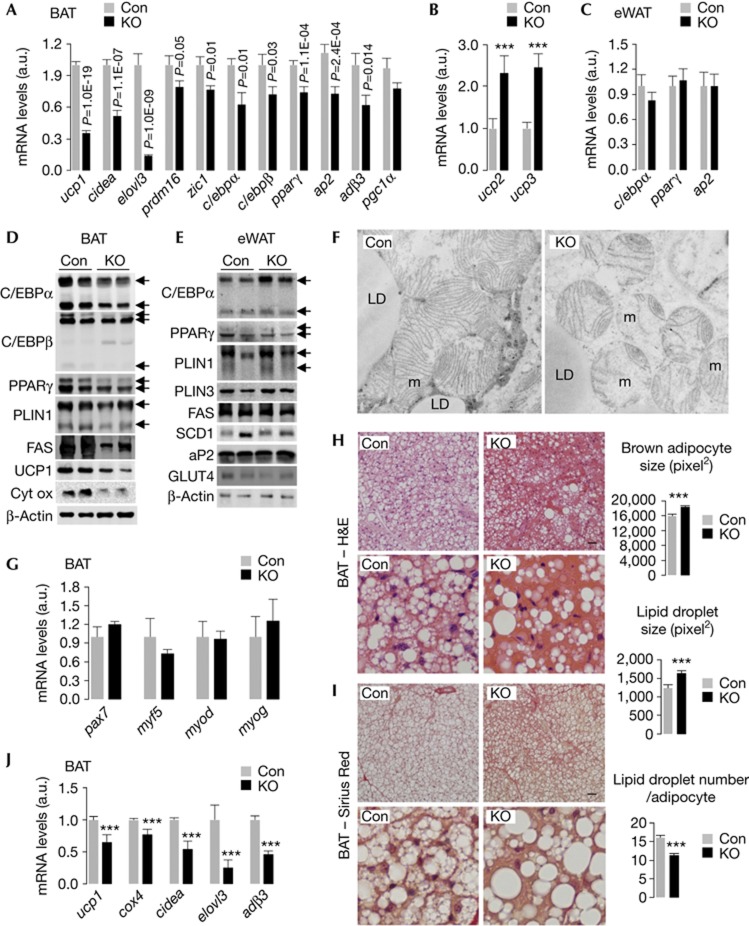
Loss of *Atg7* in Myf5+ progenitors impairs brown adipose tissue (BAT) differentiation. (**A**,**B**) mRNA for indicated genes in BAT (*n*=4) and (**C**) epididymal white adipose tissue (eWAT) (*n*=4), and (**D**) immunoblots for indicated proteins in BAT and (**E**) eWAT from 10-month (mo)-old chow diet (RD)-fed control (Con) and knock out (KO) mice. Arrows depict protein isoforms. (**F**) Electron micrographs ( × 10,000 magnification) of BAT depicting mitochondria from 4-mo-old Con and KO mice. m, mitochondria; LD, lipid droplet; n, nucleus. (**G**) mRNA levels (*n*=4) and (**H**) hematoxylin and eosin (H&E) and (**I**) Sirius Red stains in BAT from 10-mo-old Con and KO mice (*n*=3–4). Average adipocyte and LD size, and LD number in BAT are shown. Scale bar, 50 μm. (**J**) BAT mRNA levels from 4-mo-old RD-fed Con and KO mice cold-challenged for 75 min (*n*=3). Values are mean±s.e. ***P<0.001.

To determine the effect of loss of MA in Myf5+ progenitors on BAT, we subjected BAT from Con and KO mice to qPCR analysis for BAT- and adipose-selective genes. KO BAT displayed decreased expression of BAT genes, *ucp1*, *cidea*, *elovl3*, *prdm16 and zic1*, and adipose genes, *c/ebpα*, *c/ebpβ*, *pparγ* and *ap2* without modifying *pgc1*α, a transcriptional coactivator of BAT genes (Fig 2A). KO BAT also displayed ∼40% reduction in adrenergic β3 receptor (*adβ3*) expression (Fig 2A), suggesting an attenuated ability to respond to catecholamines. In contrast to effects of loss of MA in aP2+ adipocytes, that is, acquisition of BAT-like features by eWAT and augmented BAT mass [8], loss of MA in Myf5+ progenitors suppressed BAT differentiation. Surprisingly, KO BAT displayed increased expression of additional UCP family members, *ucp2* and *ucp3* (Fig 2B). As heat production is UCP1 dependent [13], the significance of increased *ucp2*/*ucp3* expression remains unclear. Loss of ATG7 in Myf5+ progenitors did not modify eWAT differentiation indicated by comparable *c/ebpα*, *pparγ* and *ap2* expression in Con and KO mice (Fig 2C).

We verified that changes in mRNA expression in KO BAT correlated with protein levels. Indeed, KO BAT showed decreased levels of C/EPBα, C/EBPβ, PPARγ, fatty acid synthase (FAS), UCP1 and the mitochondrial marker cytochrome oxidase (COX) compared with Con BAT (Fig 2D). In contrast, C/EPBα, PPARγ, perilipin (PLIN)1, PLIN3, FAS, stearoyl CoA desaturase 1 (SCD1), aP2 and GLUT4 levels remained intact in eWAT from KO mice (Fig 2E), demonstrating selective impairment in BAT differentiation. Electron microscopic analyses of KO BAT verified decreased mitochondrial number and size with regions of mitochondrial destruction between areas of preserved mitochondria (Fig 2F and supplementary Fig S2A online).

As Myf5+ progenitors give rise to BAT and SKM, we asked whether loss of MA in Myf5+ progenitors skewed the differentiation of these cells towards SKM. To test this, Con and KO BAT were analyzed for factors regulating muscle differentiation, that is, pax7 and pax3 that control the population of proliferative myogenic myf5+ cells, myf5, myod (myoblast determination protein) and myog (myogenin), which regulates conversion of myoblasts into myocytes [14]. Con and KO BAT had comparable *pax7, myf5, myod* and *myog* expression (Fig 2G), while *pax3* remained undetectable (not shown). To analyze the fate of BAT derived from *Atg7*^*−/−*^ Myf5+ cells, Con and KO BAT were subjected to hematoxylin and eosin (H&E) staining, which revealed intense eosinophilic cytoplasm, increased LD and adipocyte size, and decreased LD number/cell in KO BAT indicating a departure from the typical features of BAT (Fig 2H). In fact, Sirius red (Fig 2I; supplementary Fig S2B online) and Trichrome blue staining (supplementary Fig S2C online) revealed interspersed fibrotic areas in KO BAT, particularly at tissue septa. Although, comparable collagen gene (*col1a1* or *col3a1*) expression was detected in Con and KO BAT, increased *col6a1* expression confirmed fibrotic changes in KO BAT (supplementary Fig S2D online).

Loss of MA in Myf5+ progenitors also impacted cold-induced BAT gene expression. BAT from cold-exposed (∼4 °C for 75 min) KO mice failed to upregulate *ucp1*, *cox4*, *cidea*, *elovl3* and *adβ3* genes to levels achieved by Con (Fig 2J). KO mice were also deficient in their ability to reduce LD content in BAT indicating impaired lipid utilization (supplementary Fig S2E online).

### *Atg7* in Myf5+ cells in early BAT development

To determine the time frame when MA is required for precursor cells to differentiate into BAT, we examined the effect of loss of MA on BAT differentiation in E16.5 embryos, post-natal day 6 pups and adults. H&E stains revealed slightly smaller brown adipocyte precursors in KO embryos (supplementary Fig S3A online), and comparable, albeit low, UCP1 levels (supplementary Fig S3B online) and LD content (supplementary Fig S3A online) in precursors from Con and KO embryos. In contrast, post-natal *Atg7*^*−/−*^ BAT displayed reduced UCP1-positivity (supplementary Fig S3C online) and increased *ucp2* and *ucp3* expression (supplementary Fig S3D online), as observed in adult KO mice (Fig 2B). Furthermore, day 6 *Atg7*^*−/−*^ BAT showed altered mitochondrial morphology, that is, dilated intra-mitochondrial space and distorted mitochondrial cristae (supplementary Fig S3E online), decreased β-oxidation rates (supplementary Fig S3F online), and increased LD content (supplementary Fig S3G online), suggesting that MA is required in the early steps of BAT development, that is, after the E16.5 stage.

To determine whether acutely inhibiting MA impacts BAT differentiation in adult mice, we injected BAT of *Atg7*^Flox/Flox^ mice with Cre-expressing adenoviruses (Cre AdV) or an empty vector, and mice were killed after 5 days following an acute cold stress. Cre AdV injections decreased BAT *Atg7* mRNA by ∼30% (supplementary Fig S3H online) possibly from reduced accessibility of viruses into the entire BAT pad. This acute reduction of *Atg7* expression decreased *ucp1* and *elovl3* expression (supplementary Fig S3I online) without modifying *ucp2* or *ucp3* expression (supplementary Fig S3J online). Bodipy stains from cold-exposed Cre AdV-injected mice revealed increased LD content (supplementary Fig S3K online) as observed in KO mice (supplementary Fig S2E online), suggesting that in addition to its role in early BAT development, MA controls BAT differentiation and lipid metabolism during adulthood. It is thus likely that post-developmental changes in MA, such as with age [7], will alter BAT differentiation and lipid metabolism.

### KO mice exhibit increased body temperature

**Figure 3:**
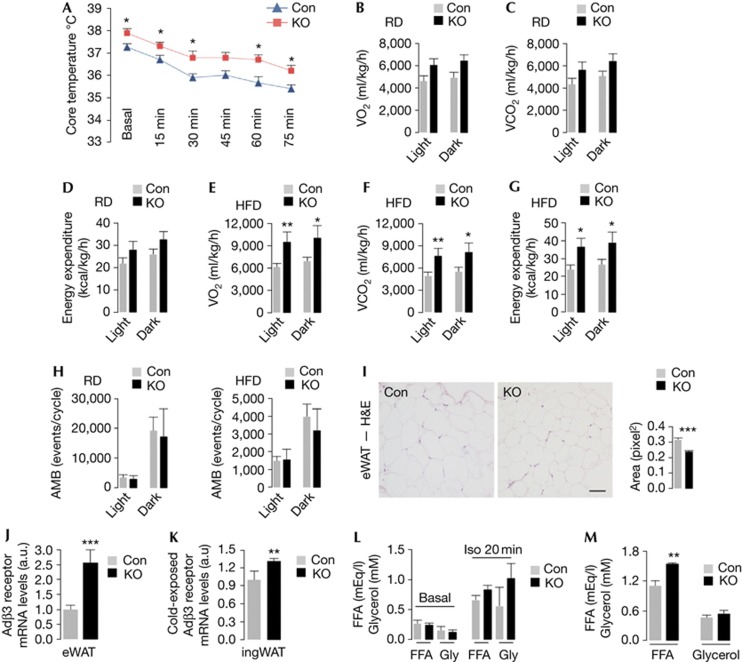
Loss of *Atg7* in Myf5+ progenitors increases energy expenditure. (**A**) Body temperature before (Basal) and during cold exposure (*n*=7–8). (**B**) Oxygen consumption (VO_2_), (**C**) carbon dioxide production (VCO_2_) and (**D**) energy expenditure in 10-month (mo)-old chow diet (RD)-fed control (Con) and knock out (KO) mice (*n*=4). (**E**) VO_2_, (**F**) VCO_2_ and (**G**) energy expenditure in 10-mo-old high-fat diet (HFD)-fed Con and KO mice (*n*=3–5). (**H**) Ambulation (AMB) in 10-mo-old RD-fed (*n*=4) and HFD-fed Con and KO mice (*n*=3–5). (**I**) Hematoxylin and eosin (H&E)-stained epididymal white adipose tissue (eWAT) from 6-mo-old RD-fed Con and KO mice. Quantification for cell size is shown (*n*=3). Scale bar, 50 μm. (**J**) Adβ3 mRNA in eWAT from 10-mo-old RD-fed Con and KO mice (*n*=4), and in (**K**) inguinal (ingWAT) from 4-mo-old cold-challenged RD-fed Con and KO mice (*n*=3). (**L**) Serum-free fatty acid (FFA) and glycerol (Gly) from 4- to 6-mo-old Con and KO mice untreated (basal) or treated with isoproterenol (Iso) i.p. for 20 min (*n*=4–6), and (**M**) from 4-mo-old RD-fed cold-challenged (75 min) mice (*n*=3–4). Values are mean±s.e. *P<0.05, **P<0.01, ***P<0.001.

To test the physiological outcome of impaired BAT differentiation, Con and KO mice were subjected to core body temperature analyses. Surprisingly, despite abnormalities in the molecular signature of BAT, KO mice maintained higher body temperature at basal conditions and during cold exposure (Fig 3A). To explore the mechanism for increased body temperature, we asked whether constitutive increases in energy expenditure raised body temperature in KO mice. Indeed, RD-fed KO mice displayed a trend towards increased oxygen (O_2_) consumption (Fig 3B; supplementary Fig S4A online), increased carbon dioxide (CO_2_) production (Fig 3C; supplementary Fig S4B online) and elevated energy expenditure (Fig 3D). RD-fed KO mice modestly decreased their respiratory exchange ratio (RER) during early dark cycle (supplementary Fig S4C online), suggesting a preference for fat oxidation to support early dark cycle activity. In contrast, HFD-fed KO mice displayed significantly increased rates of O_2_ consumption (Fig 3E; supplementary Fig S4D online), CO_2_ production (Fig 3F; supplementary Fig S4E online), and energy expenditure (Fig 3G) and persistently decreased RER (supplementary Fig S4F online) indicating sustained fat oxidation during both cycles. Higher energy expenditure did not occur from increased locomotor activity (Fig 3H), in fact, HFD-fed KO mice displayed decreased dark cycle *z* axis movements compared with Con mice (supplementary Fig S4G online).

As KO mice exhibited smaller eWAT pads and reduced RER, we asked whether constitutive increases in WAT lipolysis provided the lipid fuel to sustain higher energy expenditure rates in KO mice. Indeed, KO mice displayed smaller white adipocytes (Fig 3I) and ∼2.5-fold increase in *adβ3* expression (Fig 3J) in a compensatory response to maintain adrenergic signaling. Cold-exposed KO mice also increased their *adβ3* expression in ingWAT by ∼30% (Fig 3K). Although Con and KO mice showed equivalent basal serum-free fatty acid (FFA) and glycerol levels (Fig 3L), KO mice exhibited modest increases in circulating FFA and glycerols in response to intraperitoneal (i.p.) isoproterenol (Fig 3L), and significantly elevated serum FFA following cold exposure (Fig 3M). These results allow us to speculate that WAT lipolysis-driven increases in FFA availability/oxidation probably contribute to raised body temperature in KO mice.

### ‘Beige’ cells/BAT increase energy expenditure in KO mice

**Figure 4:**
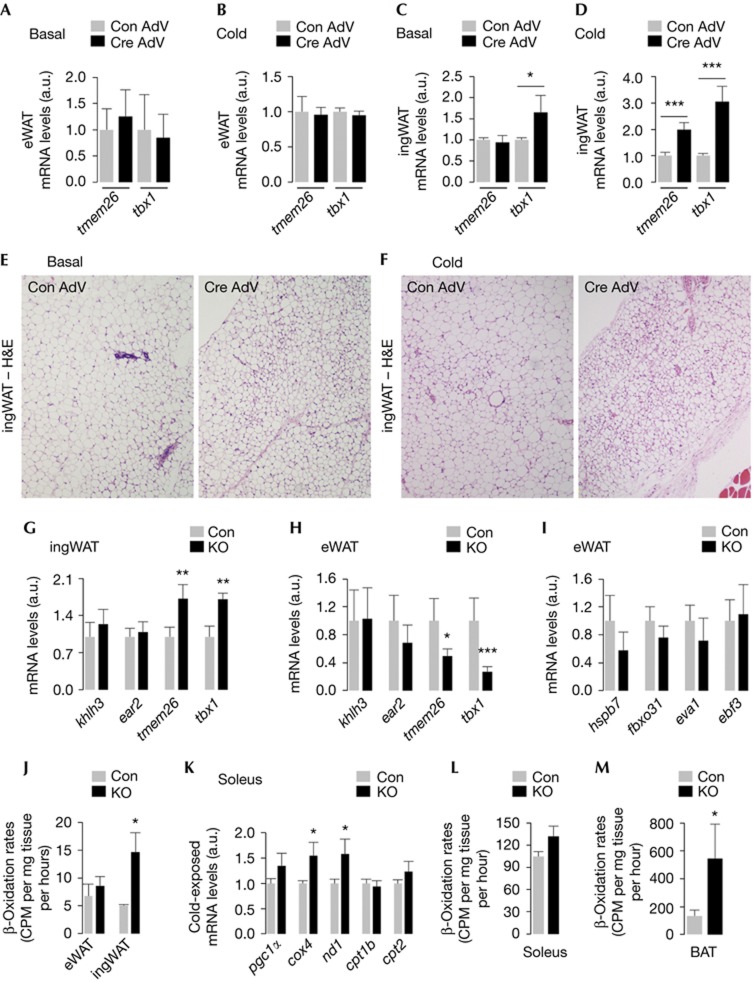
Beige cells in inguinal white adipose tissue (ingWAT) and brown adipose tissue (BAT) contribute to increased energy expenditure in knock out (KO) mice. (**A**,**B**) *Tmem26* and *tbx1* mRNA levels in epididymal eWAT, and (**C**,**D**) ingWAT, and (**E**,**F**) hematoxylin and eosin (H&E)-stained ingWAT section from *Atg7*^Flox/Flox^ mice (4 months (mo)) injected in BAT with empty (Con AdV) or Cre-expressing (Cre AdV) adenoviruses and cold-exposed or not (basal) for 75 min (*n*=4–5). (**G**) mRNA for indicated genes in ingWAT from 4-mo-old cold-exposed Con and KO mice (*n*=4). (**H,I**) mRNA levels in eWAT from 10-mo-old Con and KO mice (*n*=4). (**J**) β-oxidation in eWAT and ingWAT from 4-mo-old Con and KO mice (*n*=4). (**K**) mRNA levels in soleus from 4-mo-old cold-exposed Con and KO mice (*n*=3–6). (**L**) β-oxidation in soleus and (**M**) BAT from 4-mo-old Con and KO mice (*n*=4). Values are mean±s.e. *P<0.05, **P<0.01, ***P<0.001.

To identify the tissues that oxidized WAT-derived FFA in KO mice, we asked whether defective MA in BAT triggered ‘Beige’ cell [9] development in WAT. Acute depletion of ATG7 in BAT (via Cre AdV) did not modify basal or cold-induced expression of ‘Beige’ genes, *tmem26* or *tbx1*, in eWAT (Fig 4A,B). In contrast, ATG7 depletion led to ∼1.5-fold increase in basal *tbx1* expression in ingWAT (Fig 4C) and an approximately two- to threefold increase in *tmem26* and *tbx1* expression following cold exposure (Fig 4D). H&E stains of ingWAT from Cre AdV-injected mice confirmed presence of multi-loculated brown adipocyte-like cells (Fig 4E) that increased with cold exposure (Fig 4F; supplementary Fig S5A online).

To determine whether KO mice displayed ‘Beige’ cell development, we subjected WAT from Con and KO mice to qPCR analyses for ‘Beige’ genes [9]. As expected, we observed significantly increased expression of *tmem26* and *tbx1* in ingWAT (Fig 4G) but not eWAT (not shown) from 4-month (mo)-old cold-exposed KO mice. In fact, eWAT from 10-mo-old KO mice displayed decreased basal *tmem26* and *tbx1* expression (Fig 4H), while those of brown adipocyte genes, *hspb7*, *fbxo31*, *eva1* and *ebf3* [9], remained intact (Fig 4I). Given that small subsets of adipocyte progenitors in WAT express *myf5* [12], we speculate that loss of *Atg7* in a pool of eWAT-resident Myf5+ cells impacted ‘Beige’ cell development in eWAT, while ‘Beige’ cells in iWAT possibly originate from redundant lineages and thus remained intact. In consistency with ‘Beige’ cell development, ingWAT but not eWAT, from KO mice displayed approximately twofold increase in β-oxidation rates (Fig 4J and model summarized in supplementary Fig S7 online).

As SKM participates in thermogenesis [15], we asked whether FFA oxidation in SKM contributed to increased energy expenditure in KO mice. Despite comparable COX levels in various SKM groups from Con and KO mice (not shown), soleus from KO mice displayed higher COX levels (supplementary Fig S5B online). Soleus (Fig 4K), and not gastrocnemius (GA; supplementary Fig S5C online), from cold-exposed KO mice displayed increased *cox4*, *nd1* (subunit of NADH dehydrogenase) and *pgc1α* expression, while *cpt1b* and *cpt2* (fatty acid translocase) or *ucp2* and *ucp3* (supplementary Fig S5D online) remained comparable to Con. Increased β-oxidation in soleal explants verified their contribution to increased energy expenditure in KO mice (Fig 4L).

Equivalent β-oxidation rates in liver (supplementary Fig S5E online) from Con and KO mice excluded its role in increasing energy expenditure. Surprisingly, BAT from KO mice displayed increased β-oxidation compared with Con mice (Fig 4M). Despite the apparent defect in utilizing intrinsic lipid stores (supplementary Fig S2E online), *Atg7*^*−/−*^ BAT from adult mice maintained higher β-oxidation rates, in all likelihood, from WAT-derived FFA (modeled in supplementary Fig S7 online). Indeed, in contrast to reduced β-oxidation in KO BAT from pups (supplementary Fig S3F online), *Atg7*^*−/−*^ BAT from adult mice displayed increased β-oxidation (Fig 4M) in a likely compensatory mechanism to meet thermogenic requirements in adults.

### Smaller myofibers and glucose intolerance in KO mice

**Figure 5:**
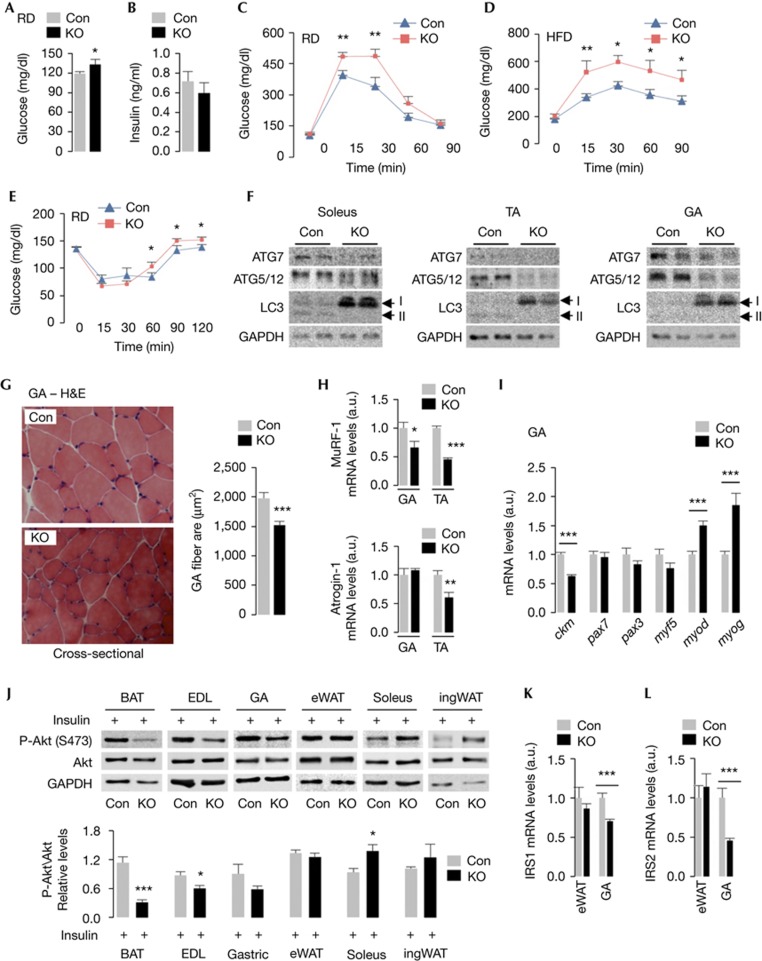
Knock out (KO) mice show reduced myofiber size and impaired glucose clearance. (**A**) Blood glucose in 6- to 10-month (mo)-old mice (*n*=12–15), and (**B**) serum insulin levels in 10-mo-old fed control (Con) and KO mice (*n*=6). (**C**) Glucose tolerance tests in 10-mo-old chow diet (RD)-fed (*n*=5), and (**D**) in 10- to 12-mo-old high-fat diet (HFD)-fed Con and KO mice (*n*=4–9). (**E**) Insulin tolerance test in 10-mo-old RD-fed Con and KO mice (*n*=5). (**F**) Immunoblots for indicated proteins in skeletal muscle from 10-mo-old Con and KO mice. (**G**) Hematoxylin and eosin (H&E)-stained gastrocnemius (GA) sections from 6-mo-old Con and KO mice. Myofiber cross-sectional area (μm^2^) is shown (*n*=3). (**H**) Expression of MuRF-1 and Atrogin-1 in GA and TA (*n*=4) and (**I**) myogenic genes in GA from 10-mo-old Con and KO mice (*n*=6). (**J**) Immunoblots for indicated proteins in tissues from 4-mo-old Con and KO mice (*n*=4). (**K**) mRNA for IRS1, (**L**) IRS2 in epididymal white adipose tissue (eWAT) and GA from 10-mo-old Con and KO mice (*n*=6). Values are mean±s.e. *P<0.05, **P<0.01, ***P<0.001.

Despite increases in energy expenditure, KO mice remained hyperglycemic (Fig 5A), euinsulinemic (Fig 5B), and displayed defective glucose clearance (Fig 5C,D) and insulin insensitivity (Fig 5E). Since MA maintains SKM mass [16] and glucose homeostasis [17], we asked whether loss of MA in Myf5+ progenitors affected myofiber size and, in turn, glucose homeostasis. In consistency with reduced MA in EDL (Fig 1B); soleus, TA and GA from KO mice also displayed defects in MA (Fig 5F). KO mice presented with reduced GA myofiber cross-sectional area by ∼25% (Fig 5G), absent centralized myonuclei (Fig 5G) and reduced expression of atrophy markers, MuRF-1 and Atrogin-1 (Fig 5H), indicating absence of SKM degeneration [16]. Reduced myofiber size probably resulted from defective SKM differentiation, indicated by decreased expression of differentiated SKM marker, creatine kinase muscle and raised levels of *myod* and *myog* (Fig 5I), while *pax7*, *pax3* and *myf5* remained intact. Embryonic loss of Myf5+ cells fails to suppress myogenesis, suggesting significant contributions to muscle development from Myf5-independent lineages [18]. Indeed, Myf5+ cells contribute to adult myonuclei by ∼50% [18], and consequently, loss of *Atg7* in Myf5+ progenitors modestly affected myocyte size supporting the previously described contribution to SKM development from both Myf5+ and Myf5-independent lineages [18]. The percentage and/or selectivity of myocytes that show defective autophagy in each SKM group following loss of *Atg7* in Myf5+ progenitors remains to be seen.

To identify the tissues contributing to glucose intolerance, Con and KO mice fed HFD for 2 weeks were subjected to i.p. insulin (1 U per kg of body weight per 30 min), and SKM and fat were analyzed for Akt phosphorylation (P-Akt). While BAT, EDL and GA from KO mice displayed decreased P-Akt (Fig 5J; supplementary Fig S6A online), soleus and ingWAT (Fig 5J; supplementary Fig S6A online) presented with increased P-Akt. Consequently, 2-deoxyglucose uptake assays revealed modest increases in glucose uptake by soleus (supplementary Fig S6B online). Intriguingly, loss of *Atg7* in Myf5+ progenitors decreased *irs1* and *irs2* expression in GA without modifying those in eWAT (Fig 5K,L) or affecting insulin receptor expression in SKM or eWAT (supplementary Fig S6C online). Changes in *irs1* and *irs2* expression probably impacted glucose clearance in KO mice, although our findings cannot distinguish whether decreased *irs1/irs2* expression occurred from defective SKM differentiation or from loss of MA *per se*. It remains possible that persistently raised mitochondrial oxidation introduced oxidative changes in SKM or BAT, which disrupted insulin signaling.

Whether the overall phenotype of KO mice is an effect of deficient MA or due to loss of a possible MA-independent function of ATG7 remains to be elucidated. Furthermore, how ATG7 in Myf5+ progenitors controls differentiation of progenitors into adipocytes remains unknown. It is possible that MA promotes differentiation of progenitors via its ability to modulate cellular energetic needs or eliminate regulatory proteins and/or maintain quality control. Alternatively, the established role for insulin in driving adipogenesis, and the effect of loss of MA on insulin signaling might explain why loss of *Atg7* impacts adipose differentiation. Aging associates with reduced ATG7 levels [7] and it is likely that MA failure in Myf5+ progenitors with age interferes with tissue differentiation, which contributes to metabolic defects and sarcopenia. Maintaining MA activity in Myf5+ progenitors might help prevent abnormalities in glucose metabolism and/or sarcopenia observed with age.

## Methods

**Chemicals and antibodies**. Antibodies against ATG7, FAS, GLUT4, IRS1, LC3, PPARγ and SCD1 (Cell Signaling Technology, Danvers, MA); aP2, C/EBPα and C/EBPβ (Santa Cruz Biotechnology, Santa Cruz, CA); COX (Mitosciences, Eugene, OR); PLIN1 (Progen, Heidelberg, Germany); PLIN3 (Prosci Inc, Poway, CA); ATG5 (Novus Biologicals, Littleton, CO); and UCP1, Actin and GAPDH (Abcam, Cambridge, MA) were used in this study. Isoproterenol, glucose and insulin were purchased from Sigma-Aldrich (St Louis, MO).

**Animals and cells**. Myf5-Cre mice were obtained from Jackson Laboratories, Bar Harbor, ME [11], and *Atg7*^Flox/Flox^ mouse was a gift from Drs M Komatsu and K Tanaka (Tokyo Metropolitan Institute of Medical Science, Tokyo, Japan) [10]. Studies were performed in KO mice and their littermate controls that lacked *cre*. Mice were fed regular chow (no. 5058; Lab Diet) or HFD (60% kcal in fat; D12492; Research Diets, New Brunswick, NJ) and maintained in 12 h light/dark cycles. Genotyping was performed using established primers [10]. Mice were used under a protocol approved by the Institutional Animal Care and Use Committee.

**Adenoviral infection**. A total of 2 × 10^7^ particle-forming units of control adenovirus or Cre-expressing adenovirus (Vector BioLabs) was injected into BAT of *Atg7*^Flox/Flox^ mice. At 5 days post injection, mice were cold-challenged (4 °C/75 min) and killed.

**Core body temperature measurements**. Body temperature (°C) was measured by inserting a rectal thermometer (BIOSEB, Pinellas Park, FL) 1 cm into the rectum and allowed to stabilize for 5 s and values were recorded every 15 min. Rodents were killed if core body temperature dropped below 25 °C.

**β-Oxidation assay**. Tissues from WT and KO mice were subjected to ^14^C-oleic acid-labeled β-oxidation analysis as described [8].

**General methods**. Adipose and muscle proteins were harvested in lysis buffer containing protease and phosphatase inhibitors and subjected to western blot analysis, as described [8].

**Statistics**. Results are mean±s.e. and represent data from a minimum of three independent experiments. Groups were compared by two-tailed Student’s *t-*test. Statistical significance was defined as *P*<0.05.

Supplementary information is available at EMBO *reports* online (http://www.emboreports.org).

## Supplementary Material

Supplementary Information

Review Process File
