# Supplementary material for: Autophagy in Myf5+ progenitors regulates energy and glucose homeostasis through control of brown fat and skeletal muscle development
Source: EMBO Rep. 2013 Aug 2;14(9):795–803. doi: 10.1038/embor.2013.111 (PMC3790054; doi:10.1038/embor.2013.111)
Supplement: Supplementary Information [file embor2013111s1.pdf]

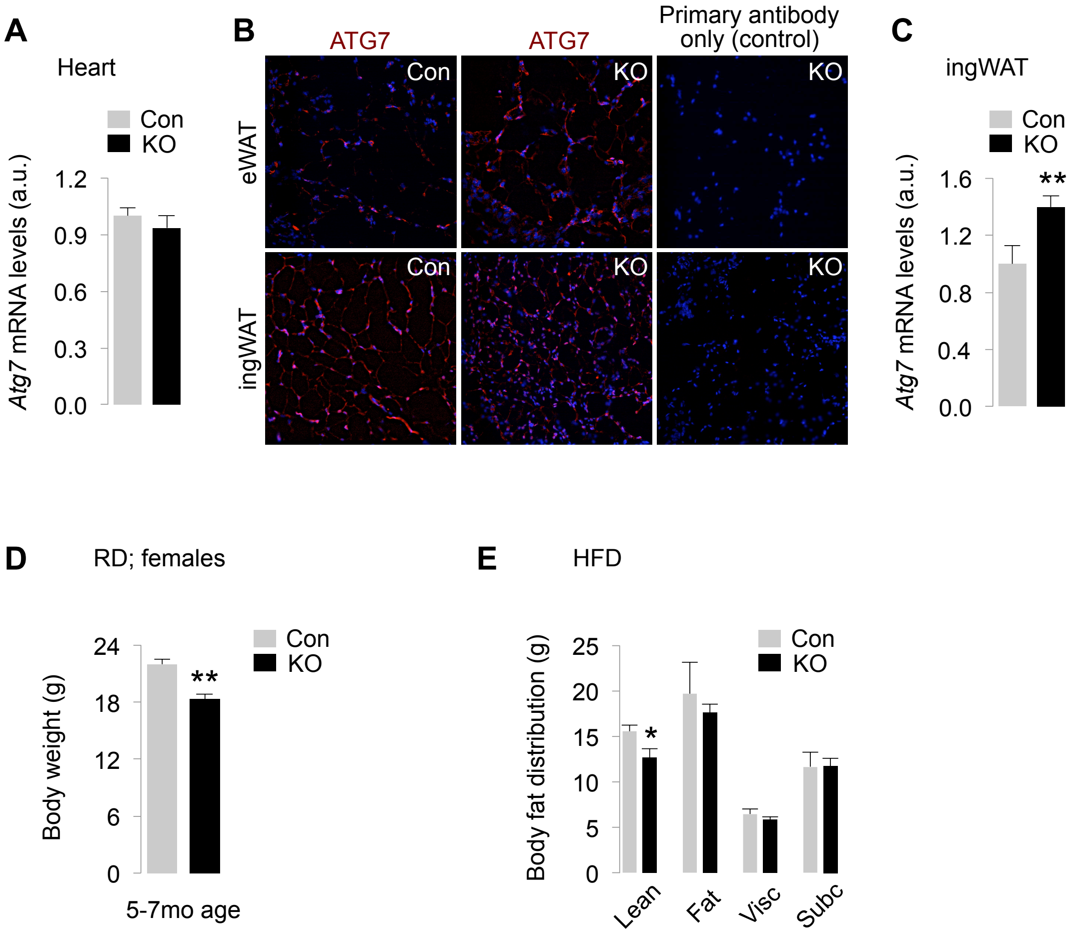
A
Heart
Con
KO
Atg7 mRNA levels (a.u.)
1.2
0.9
0.6
0.3
0.0
B
ATG7
ATG7
Primary antibody only (control)
Con
KO
KO
eWAT
ingWAT
C
ingWAT
Con
KO
Atg7 mRNA levels (a.u.)
1.6
1.2
0.8
0.4
0.0
**
D
RD; females
Body weight (g)
24
18
12
6
0
**
5-7mo age
E
HFD
Body fat distribution (g)
25
20
15
10
5
0
*
Lean
Fat
Visc
Subc

Fig. S1

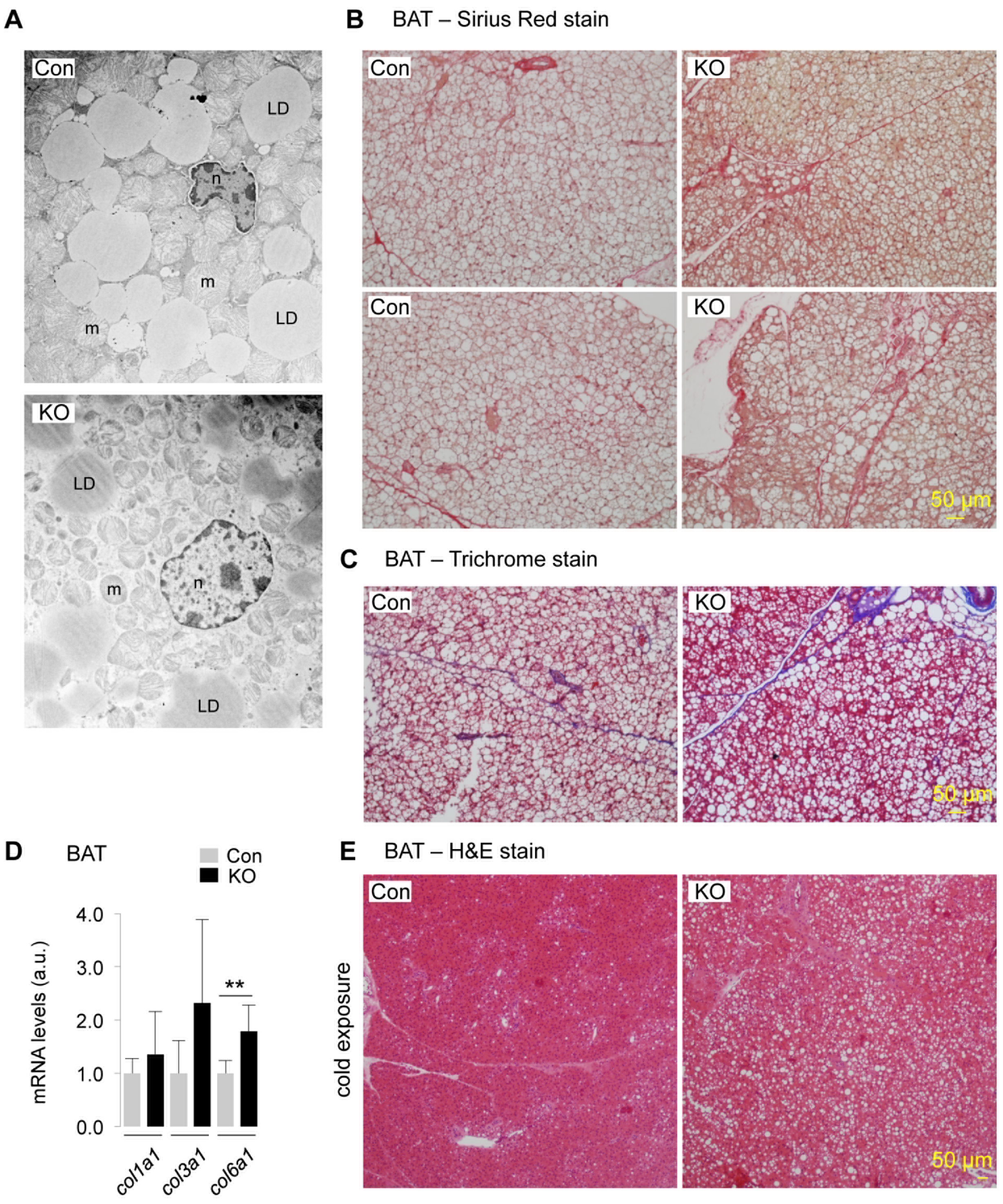
A
Con
LD
n
m
m
LD
KO
LD
m
n
LD
B
BAT – Sirius Red stain
Con
KO
Con
KO
50 µm
C
BAT – Trichrome stain
Con
KO
50 µm
D
BAT
Con
KO
mRNA levels (a.u.)
4.0
3.0
2.0
1.0
0.0
**
col1a1
col3a1
col6a1
E
BAT – H&E stain
cold exposure
Con
KO
50 µm

Fig. S2

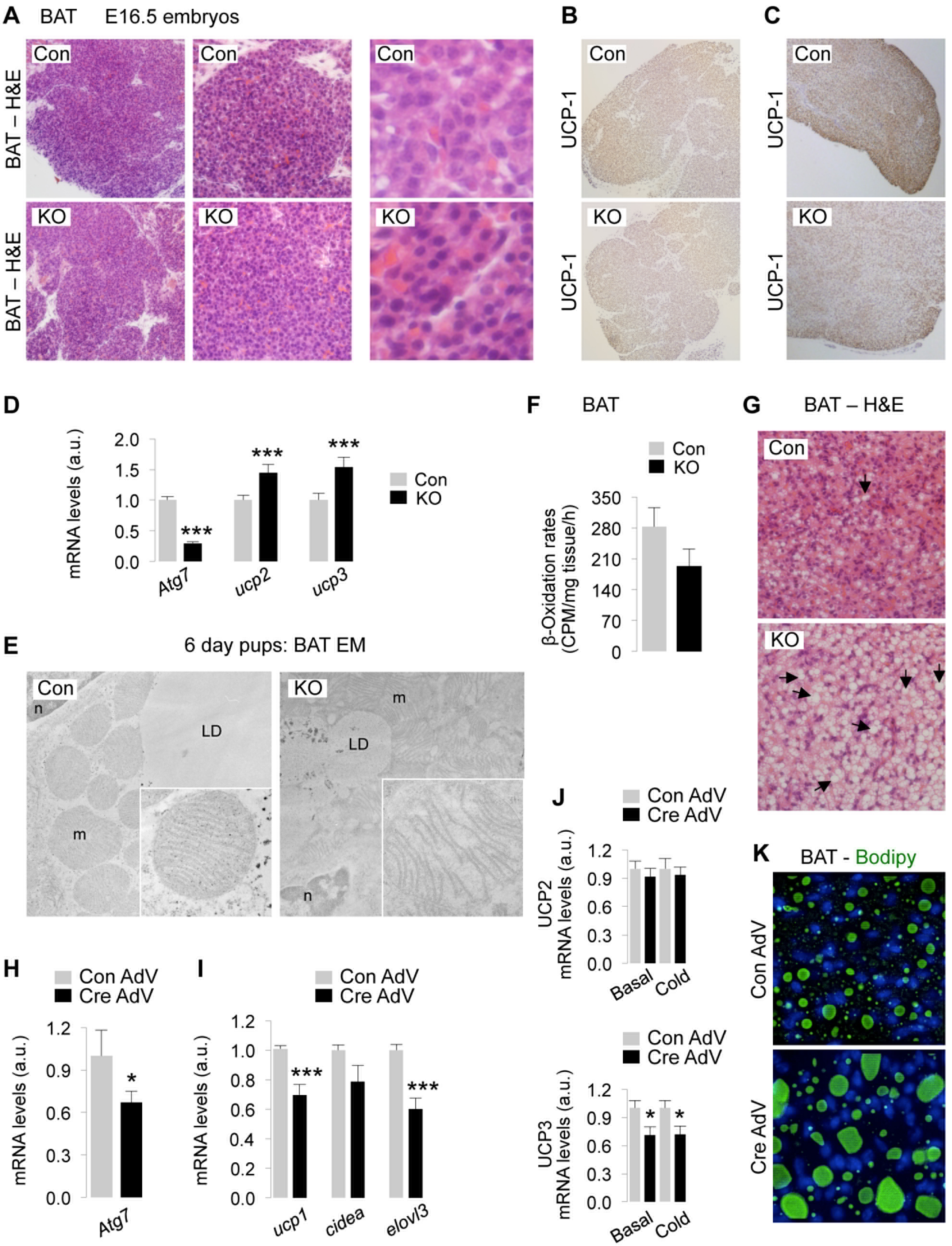
A BAT E16.5 embryos
Con
KO
BAT – H&E
B
C
UCP-1
D
mRNA levels (a.u.)
2.0
1.5
1.0
0.5
0.0
Atg7
ucp2
ucp3
***
Con
KO
E
6 day pups: BAT EM
n
LD
m
F
BAT
β-Oxidation rates (CPM/mg tissue/h)
350
280
210
140
70
0
G
BAT – H&E
H
Con AdV
Cre AdV
1.2
0.9
0.6
0.3
*
I
0.8
0.4
0.2
ucp1
cidea
elovl3
J
UCP2 mRNA levels (a.u.)
UCP3 mRNA levels (a.u.)
Basal
Cold
K
BAT - Bodipy

Fig. S3

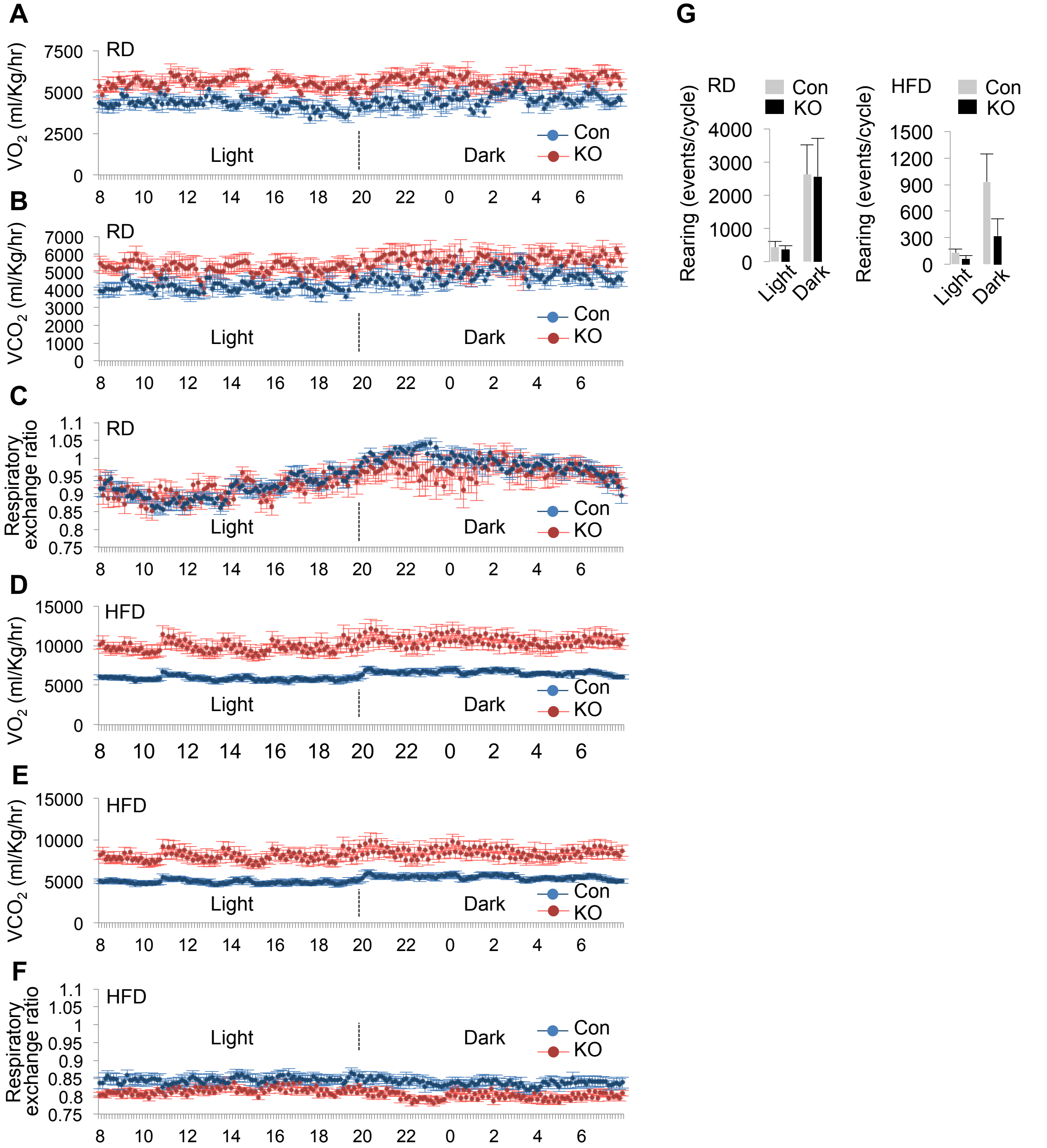

A
RD
VO2 (ml/Kg/hr)
Light
Dark
Con
KO
B
VCO2 (ml/Kg/hr)
C
Respiratory exchange ratio
D
HFD
E
F
G
Rearing (events/cycle)

Fig. S4

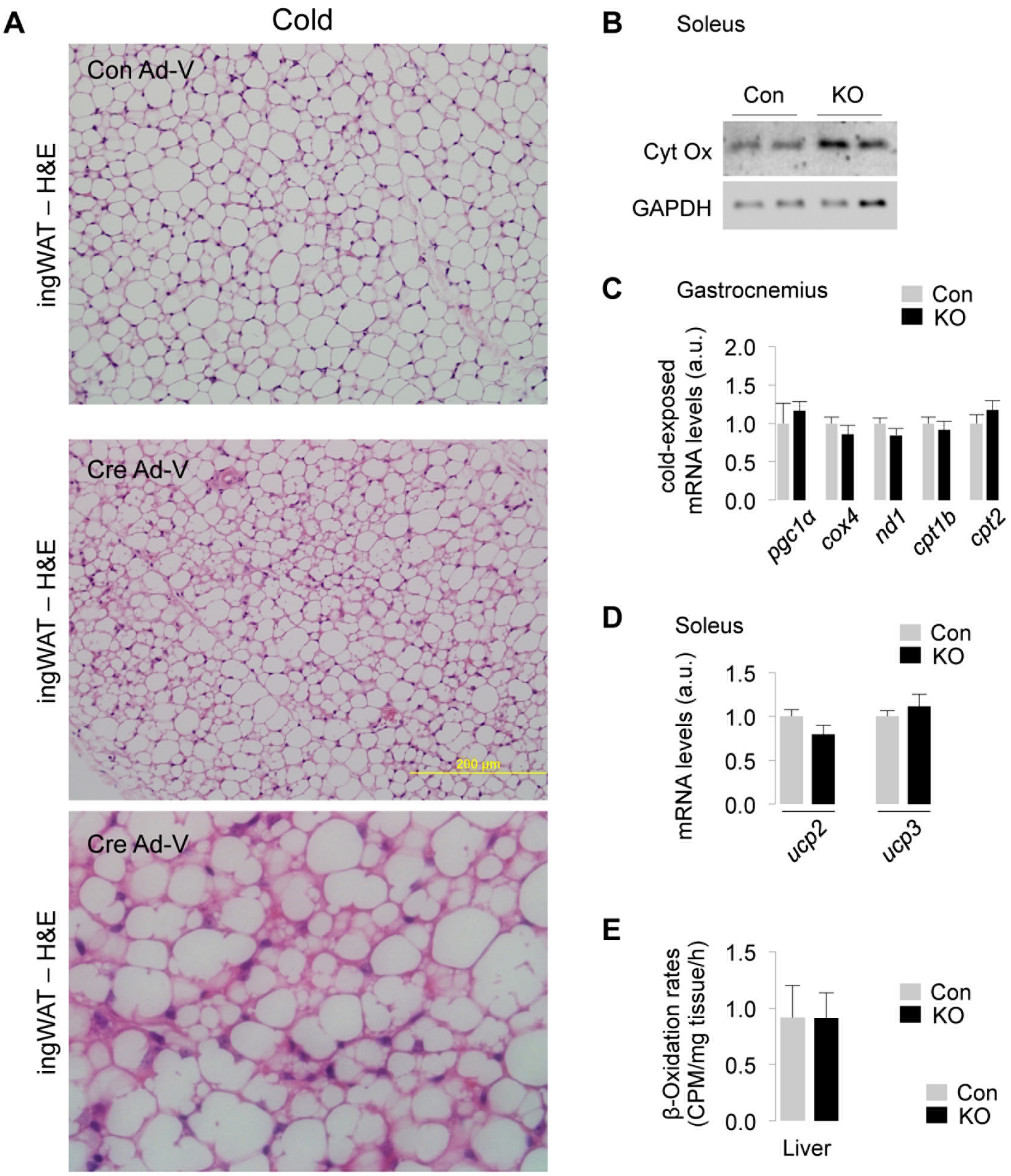

A
Cold
Con Ad-V
Cre Ad-V
Cre Ad-V
ingWAT – H&E
ingWAT – H&E
ingWAT – H&E
200 μm
B
Soleus
Con
KO
Cyt Ox
GAPDH
C
Gastrocnemius
Con
KO
cold-exposed mRNA levels (a.u.)
2.0
1.5
1.0
0.5
0.0
pgc1α
cox4
nd1
cpt1b
cpt2
D
Soleus
Con
KO
mRNA levels (a.u.)
1.5
1.0
0.5
0.0
ucp2
ucp3
E
β-Oxidation rates (CPM/mg tissue/h)
1.5
1.0
0.5
0.0
Con
KO
Con
KO
Liver

Fig. S5

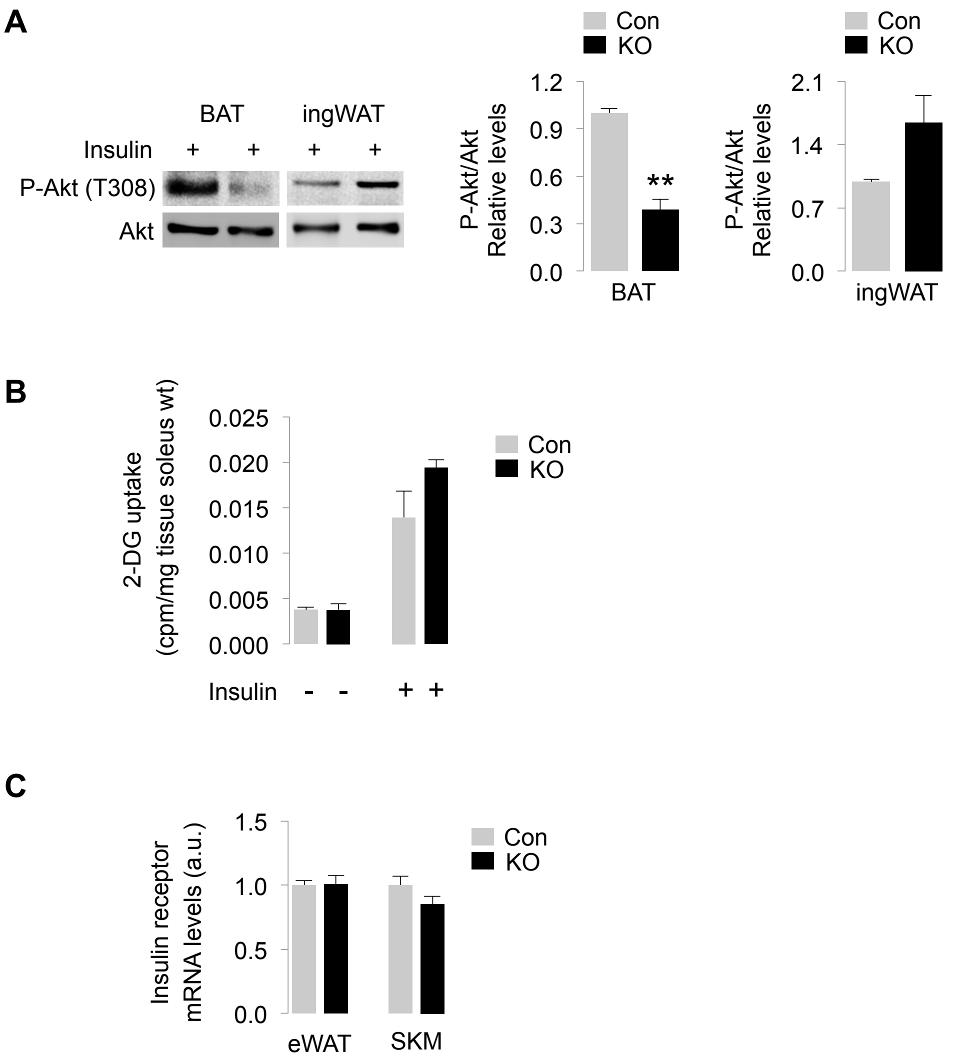
A
Con
KO
BAT
ingWAT
Insulin
+
+
+
+
P-Akt (T308)
Akt
P-Akt/Akt
Relative levels
1.2
0.9
0.6
0.3
0.0
**
2.1
1.4
0.7
0.0
B
2-DG uptake
(cpm/mg tissue soleus wt)
0.025
0.020
0.015
0.010
0.005
0.000
Insulin - - + +
C
Insulin receptor
mRNA levels (a.u.)
1.5
1.0
0.5
0.0
eWAT
SKM

Fig. S6

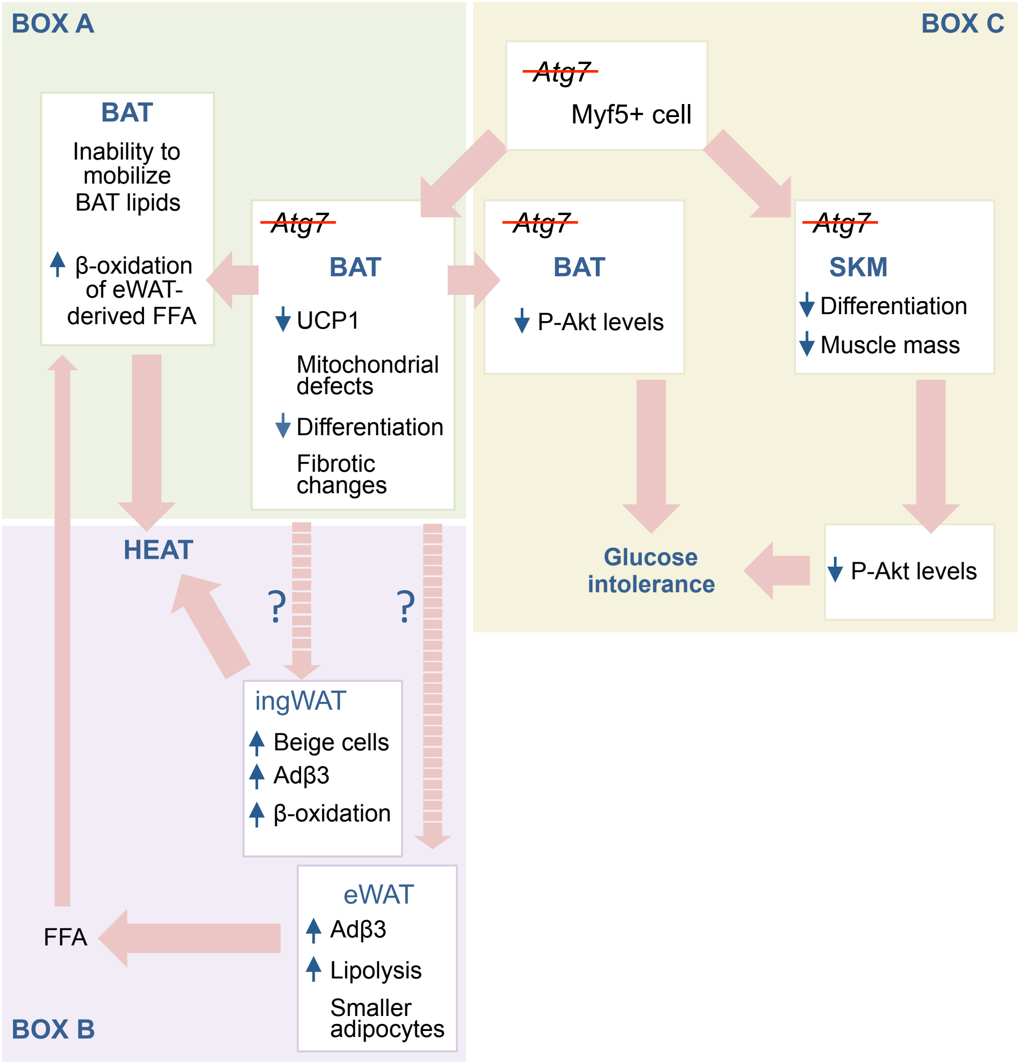
BOX A
BAT
Inability to mobilize BAT lipids
↑ β-oxidation of eWAT-derived FFA
Atg7
BAT
↓ UCP1
Mitochondrial defects
↓ Differentiation
Fibrotic changes
HEAT
?
?
ingWAT
↑ Beige cells
↑ Adβ3
↑ β-oxidation
eWAT
↑ Adβ3
↑ Lipolysis
Smaller adipocytes
FFA
BOX B
BOX C
Atg7
Myf5+ cell
Atg7
BAT
↓ P-Akt levels
Atg7
SKM
↓ Differentiation
↓ Muscle mass
Glucose intolerance
↓ P-Akt levels

Fig. S7

Supplementary Information

Fig S1 (A) *Atg7* expression in Heart from 3 month (mo) old control (Con) and KO mice. **(B)** ATG7 immunostains in epididymal WAT (eWAT) and inguinal WAT (ingWAT) from 3 mo old Con and KO mice. ATG7 primary antibody-only negative controls are also shown. **(C)** *Atg7* expression in ingWAT from 3 mo old Con and KO mice. **(D)** Body weights of 5-7 mo old RD-fed control and KO female mice (n=5), and **(E)** CT analyses for lean- and fat mass, and body fat distribution in visceral (Visc) and subcutaneous (Subc) fat depots in 10 mo HFD-fed old Con and KO mice (n=4). Values are mean+SE. *p<0.05, **p<0.01 as compared to Con.

Fig S2 (A) EM of BAT sections (2,000X magnification) from Con and KO mice. LD – lipid droplet, m – mitochondria, n – nucleus. **(B)** Sirius Red stains, **(C)** trichrome stains, and **(D)** *col1a1*, *col3a1*, *col6a1* expression in BAT from 10 mo old RD-fed Con and KO mice (n=4). **(E)** Hematoxylin and Eosin (H&E) stains in BAT from 75min cold-exposed, 3-6 mo old RD-fed control and KO mice. Values indicate mean+SE. **p<0.01 as compared to Con.

Fig S3 (A) Hematoxylin and eosin (H&E)-stained brown adipocyte precursors from E16.5 Con and KO embryos. 20X (left panel) and 40X (right panel) images are shown. **(B)** UCP1 immunostains in BAT from E16.5 Con and KO embryos and in **(C)** 6 day-old Con and KO pups. **(D)** *Atg7*, *ucp2*, and *ucp3* expression in 6 day old Con and KO pups (n=4). **(E)** Electron micrographs (5,000X magnification, 10,000X inserts) of BAT depicting mitochondria from 6 day old Con and KO pups, m – mitochondria, LD – lipid droplet, n – nucleus. **(F)** Rates of β-oxidation (CPM/mg tissue/h) in BAT from 6 day old Con and KO pups (n=4). **(G)** H&E stains of BAT from 6 day old Con and KO pups. Arrows indicate LD. **(H)** mRNAs for *Atg7*, **(I)** *ucp1*, *cidea* and *elovl3*, and **(J)** *ucp2* and *ucp3* (n=4), and **(K)** bodipy-stained BAT sections from 4 mo old $Atg7^{Flox/Flox}$ BAT injected with empty vector (Con AdV) or Cre-expressing adenoviruses (Cre

AdV) for 5 days followed by acute cold stress (4°C/75 min). Values are mean+SE, *p<0.05, ***p<0.001.

Fig S4 (A) 24h rates of oxygen consumption (VO_2), **(B)** carbon dioxide production (VCO_2), and **(C)** respiratory exchange ratio in 10 mo old RD-fed Con and KO mice (n=4). 24h rates of **(D)** VO_2, **(E)** VCO_2, and **(F)** respiratory exchange ratio in 10 mo old Con and KO mice fed HFD for 8 mo (n=3-5). **(G)** Z-axis movement (rearing expressed as events/cycle) in 10 mo old RD- and HFD-fed control and KO mice.

Fig S5 (A) H&E stains of ingWAT from *Atg7*$^{Flox/Flox}$ mice subjected to Con AdV or Cre AdV injections and exposed to acute cold exposure. Top (Con AdV) and middle (Cre AdV) are 20X images, and lower panel is 60X image of Cre AdV-injected BAT. **(B)** Cytochrome oxidase levels in soleus from 10 mo Con and KO mice. GAPDH is the loading control. **(C)** Expression of indicated genes in gastrocnemius and **(D)** soleus from 4 mo old cold-exposed Con and KO mice (n=3-6). **(E)** Liver β-oxidation from 4 mo old Con and KO mice (n=4).

Fig S6 (A) Insulin-stimulated P-Akt (threonine (T)308) levels in BAT and ingWAT and their corresponding quantifications in 4 mo old Con and KO mice. **(B)** 2-deoxyglucose (2-DG) uptake (cpm/mg tissue weight) by insulin-treated or untreated soleus from 4 mo old Con and KO mice (n=4). **(C)** mRNA levels for insulin receptor in eWAT and SKM (gastrocnemius) from 10 month (mo) old Con and KO mice (n=6).

Fig S7 Macroautophagy (MA) in Myf5+ progenitors regulates energy and glucose homeostasis through control of brown fat and skeletal muscle development. **BOX A:** Loss of *Atg7* blocks BAT differentiation, promotes mitochondrial defects, prevents utilization of BAT-intrinsic lipids and introduces fibrotic changes in BAT. **BOX B:** Through unclear mechanisms decreased BAT differentiation triggers (i) compensatory "Beige" cell development in ingWAT and (ii) increases lipolysis in eWAT that contribute directly (increased β-oxidation in Beige cells) or indirectly (release of free fatty acids/FFA for utilization by tissues for β-oxidation) to increase body

temperature in KO mice. **BOX C:** MA-deficient SKM display reduced muscle differentiation and mass, and both BAT and SKM derived from *Atg7*$^{-/-}$ Myf5+ progenitors exhibit reduced Akt phosphorylation in response to insulin, which likely contributes to the glucose intolerance in KO mice.

Supplementary Methods

Metabolic profiling

An ECHO (Echo Medical Systems) magnetic resonance spectroscopy instrument was used for body composition determination as previously (Singh et al., 2009). For energy expenditure assessments, mice were acclimatized to individual metabolic chambers. Metabolic parameters (oxygen consumption, carbon dioxide production, and locomotor activity) were examined continuously using a CLAMS (Columbus Instruments) open-circuit indirect calorimetry for 7 days.

Histological analyses

The Histology and Comparative Pathology core at the Albert Einstein College of Medicine performed histological analyses. Paraffin-embedded sections (5µm thick) of formalin-fixed tissues were subjected to Hematoxylin and Eosin (H/E), Sirius Red and Trichrome staining. Sections were analyzed under a Nikon light microscope at the indicated magnification and quantified with ImageJ software (NIH, USA).

Fluorescence microscopy

Frozen sections (5µm thick) were fixed in 3% paraformaldehyde in PBS for 10 minutes, blocked with 3% horse serum, 1% BSA in PBS containing 0.4% triton X-100 for 1 hour at room

temperature and stained with the corresponding primary and secondary-conjugated antibodies. For lipid droplet detection, sections were incubated with BODIPY 493/503 for 20 minutes at room temperature. Mounting medium contained DAPI (4', 6-diamidino-2-phenylindole) to visualize the nucleus (Invitrogen). Images were acquired on a Leica DMI6000B microscope/DFC360FX 1.4-megapixel monochrome digital camera (Leica Microsystems, Germany).

Electron microscopy

BAT from day 6 neonates and adult WT and KO mice were subjected to electron microscopy as described (*Singh et al, JCI 2009*).

In Vitro **Insulin Stimulated Glucose Uptake in Soleus Muscle**

After dissection, split soleus muscles were preincubated in oxygenated (95% O_2, 5% CO_2) Krebs-Henseleit bicarbonate (KHB) buffer containing 2mM pyruvate, 36mM mannitol, and 0.1% bovine serum albumin (preincubation buffer) to recover for 30min at 29°C. Then soleus muscles were incubated at 29°C in oxygenated preincubation buffer with or without insulin (100 uU/ml) for 60 min. After incubation with 1mM [^{3}H] 2-deoxyglucose and 39mM [1-^{14}C] mannitol for an additional 15min, soleus muscles were freeze-clamped *in situ* and kept in -80°C until analysis.

RNA isolation and RT-PCR analyses

Total RNA was isolated using the Trizol Reagent (Invitrogen). The aqueous phase containing the RNA was loaded onto a gDNA Eliminator Spin Column (Qiagen) for elimination of genomic DNA, and RNA was isolated using the RNeasy Plus kit (Qiagen, USA) according to manufacturer's instructions. Total RNA (1µg) was reverse transcribed into cDNA using

Superscript II (Invitrogen), and quantitative RT-PCR analyses was performed using the Power SYBR® Green PCR Master Mix (Applied Biosystems, UK) on a StepOnePlus Real-Time PCR System (Applied Biosystems, UK). For each gene, values were normalized to the expression of the housekeeping gene TATA-binding protein (TBP) as described previously (*Seale et al, Nature 2008*). The mRNA expression in control mice was considered as 1 and mRNA expression in KO samples were represented as fold-change compared to expression in controls. Comparisons were only made for expression levels between the same gene in control or KO samples. All reactions were performed in triplicate. Values were expressed in arbitrary units (a.u).

The following primers were used:

Adβ3 forward (f) 5'-ggcaacctgctggtaatcat-3', reverse (r) 5'- tccactgacgtccacagttc-3'
FABP4/aP2 (f) 5'-catcagcgtaaatggggatt-3', (r) 5'-tcgactttccatcccacttc-3'
ATGL (f) 5'-atttatcccggtgtactgtggcct-3', (r) 5'-agtggcaagttgtctgaaatgccg-3'
Atrogin-1 (f) 5'- gcaaacactgccacattctctc-3', (r) 5'-cttgaggggaaagtgagacg-3'
C/EBPα (f) 5'-tggacaagaacagcaacgag-3', (r) 5'-ccttgaccaaggagctctca-3'
C/EBPβ (f) 5'-caagctgagcgacgagtaca-3', (r) 5'-agctgctccaccttcttctg-3'
Cidea (f) 5'-tgctcttctgtatcgcccagt-3', (r) 5'-gccgtgttaaggaatctgctg-3'
CKM (f) 5'-catggagaagggaggcaata-3', (r) 5'-gacgaaggcgagtgagaatc-3'
Col1a1 (f) 5'-acgggaatccatcggtca-3', (r) 5'-gacgccatcaaggtctactg-3'
Col3a1 (f) 5'-gggtttccctggtcctaaag-3', (r) 5'-cctggtttcccattttctcc-3'
Col6a1 (f) 5'-gatgagggtgaagtgggaga-3', (r) 5'-cagcacgaagaggatgtcaa-3'
COX4 (f) 5'-gccccatccctcatactttc-3', (r) 5'-gctctcacttcttccactcattct-3'
Ear2 (f) 5'-cctgtaaccccagaactcca-3', (r) 5'-cagatgagcaaaggtgcaaa-3'
Ebf3 (f) 5'-cgaaaggaccgcttttgtgg-3', (r) 5'-agtgaatgccgttgttggttt-3'
ELOVL3 (f) 5'-tccgcgttctcatgtaggtct-3', (r) 5'-ggacctgatgcaaccctatga-3'
Eva1 (f) 5'-ccacttctcctgagtttacagc-3', (r) 5'-gcattttaaccgaacatctgtcc-3'
Fbxo31 (f) 5'-aaactgcttcaccgatacagac-3', (r) 5'-accacgacgttcagcaatcc-3'
Hspb7 (f) 5'-gagcatgttttcagacgactttg-3', (r) 5'-ccgagggtcttgatgtttcctt-3'
IR (f) 5'-aaagtttgcccaaccatctg-3', (r) 5'-gtgaaggtcttggcagaagc-3'
IRS-1 (f) 5'-ccagcctggctatttagctg-3', (r) 5'-cccaactcaactccaccact-3'
IRS-2 (f) 5'-gtagttcaggtcgcctctgc-3', (r) 5'-cagctattgggaccaccact-3'

Klhl3 (f) 5’-agaattggttgctgcaatactcc-3’, (r) 5’-aaggcacagtttcaagtgctg-3’

Myf5 (f) 5’-agacgcctgaagaaggtcaa-3’, (r) 5’-gttctccacctgttccctca-3’

MyoD1 (f) 5’-agtgaatgaggccttcgaga-3’, (r) 5’-gcatctgagtcgccactgta-3’

MyoG (f) 5’-ctacaggccttgctcagctc-3’, (r) 5’-acgatggacgtaagggagtg-3’

MuRF-1 (f) 5’-acctgctggtggaaaacatc-3’, (r) 5’-cttcgtgttccttgcacatc-3’

ND1 (f) 5’-tgtggaagaagcagatgttg-3’, (r) 5’-aagacctcgcagaatgaatg-3’

Pax3 (f) 5’-aaacccaagcaggtgacaac-3’, (r) 5’-ctagatccgcctcctcctct-3’

Pax7 (f) 5’-gagttcgattagccgagtgc-3’, (r) 5’-cgggttctgattccacatct-3’

PGC1α (f) 5’-ccctgccattgttaagacc-3’, (r) 5’-tgctgctgttcctgttttc-3’

PLIN2 (f) 5’-tctcagcagggttaaagaggccaa-3’, (r) 5’-tcctggcgaattcaatcaggtgga-3’

PLIN3 (f) 5’-tcacgtgtgggacagatggtgatt-3’, (r) 5’-agcccaaccggacaaagtagttct-3’

PPARγ (f) 5’-ttttcaagggtgccagtttc-3’, (r) 5’-aatccttggccctctgagat-3’

PRDM16 (f) 5’-cagcacggtgaagccattc-3’, (r) 5’-gcgtgcatccgcttgtg-3’

TBP (f) 5´-gaagctgcggtacaattccag-3´, (r) 5´-ccccttgtacccttcaccaat-3’

Tbx1 (f) 5´-ggcaggcagacgaatgttc-3´, (r) 5´-ttgtcatctacgggcacaaag-3’

Tmem26 (f) 5´-accctgtcatcccacagag-3´, (r) 5´-tgtttggtggagtcctaaggtc-3’

UCP1 (f) 5’-actgccacacctccagtcatt-3’, (r) 5’-ctttgcctcactcaggattgg-3’

UCP2 (f) 5’-ctacaagaccattgcacgagagg-3’, (r) 5’-agctgctcataggtgacaaacat-3’

UCP3 (f) 5’-atgagttttgcctccattcg-3’, (r) 5’-ggcgtatcatggcttgaaat-3’

ZIC1 (f) 5’-tctgcttctgggaggagtgt-3’, (r) 5’-ctcccctgtgtgtgtccttt-3’

ELISA

Serum Insulin levels were assessed by the Hormone core facility at the Albert Einstein College of Medicine.

Supplementary References

Bates, S.H., Dundon, T.A., Seifert, M., Carlson, M., Maratos-Flier, E., and Myers, M.G., Jr. (2004). LRb-STAT3 signaling is required for the neuroendocrine regulation of energy expenditure by leptin. Diabetes 53, 3067-3073.

Singh, R., Kaushik, S., Wang, Y., Xiang, Y., Novak, I., Komatsu, M., Tanaka, K., Cuervo, A.M., and Czaja, M.J. (2009). Autophagy regulates lipid metabolism. Nature 458, 1131-1135.

Seale, P., Bjork, B., Yang, W., Kajimura, S., Chin, S., Kuang, S., Scime, A., Devarakonda, S., Conroe, H.M., Erdjument-Bromage, H., Tempst, P., Rudnicki, M.A., Beier, D.R., and Spiegelman, B.M. (2008). PRDM16 controls a brown fat/skeletal muscle switch. Nature 454, 961-967.
