# Supplementary figures and images for: Autophagy in Myf5+ progenitors regulates energy and glucose homeostasis through control of brown fat and skeletal muscle development
Source: EMBO Rep. 2013 Aug 2;14(9):795–803. doi: 10.1038/embor.2013.111 (PMC3790054; doi:10.1038/embor.2013.111)

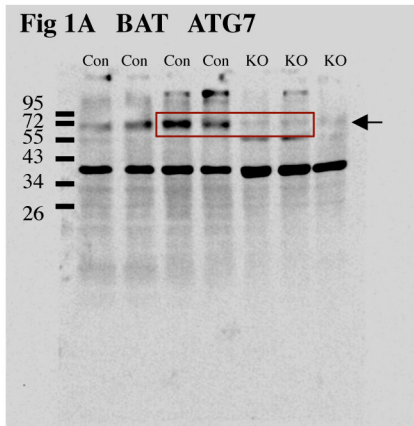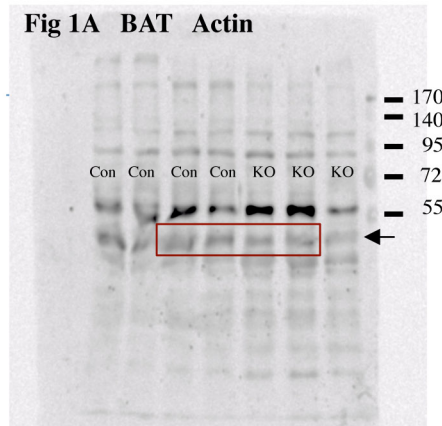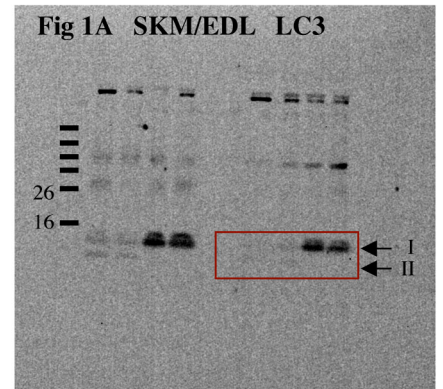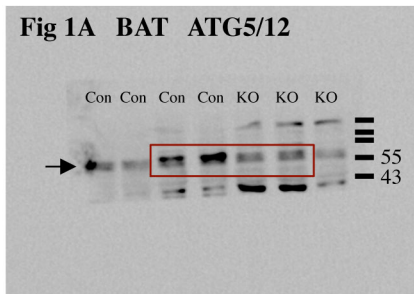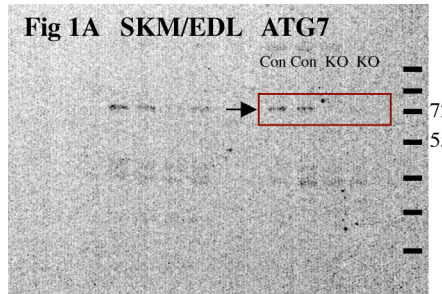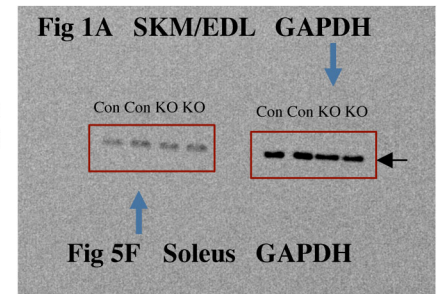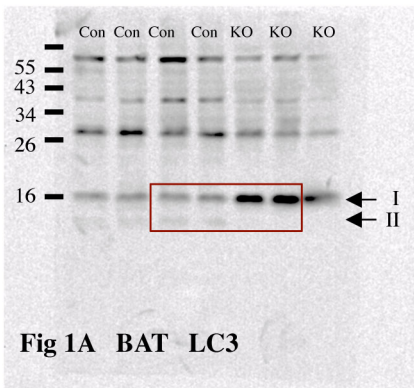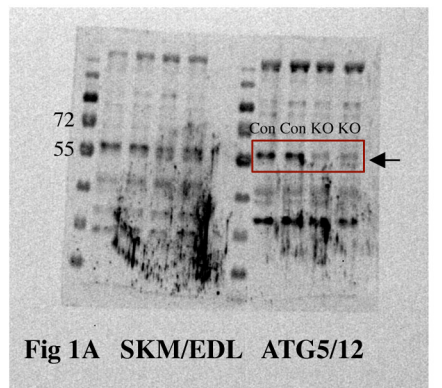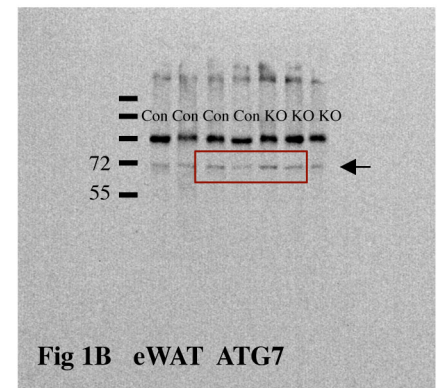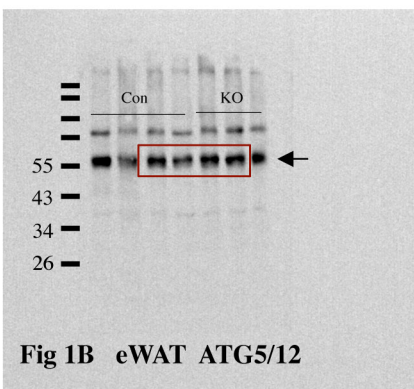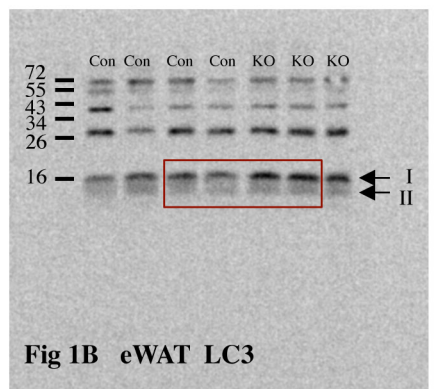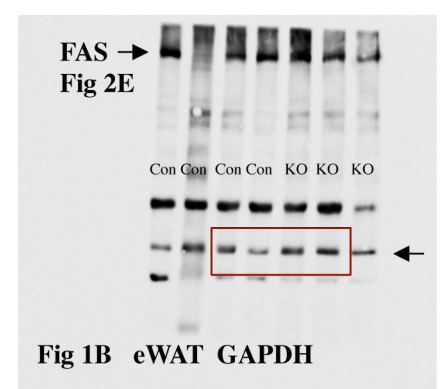

**Fig 1A and 1B**

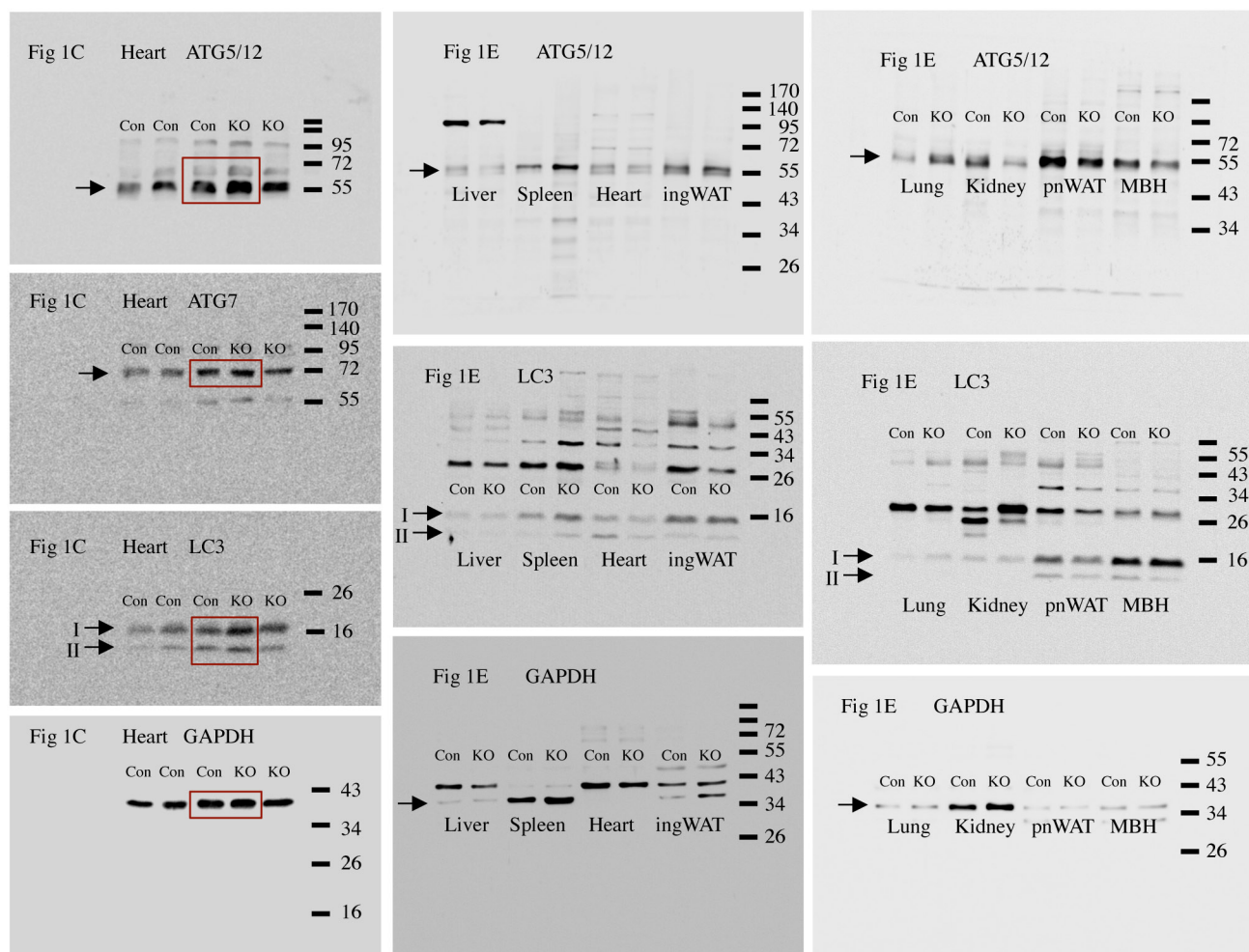

**Fig 1C and 1E**

Supplement: Source data for Figure 1 [file embor2013111df1.pdf]

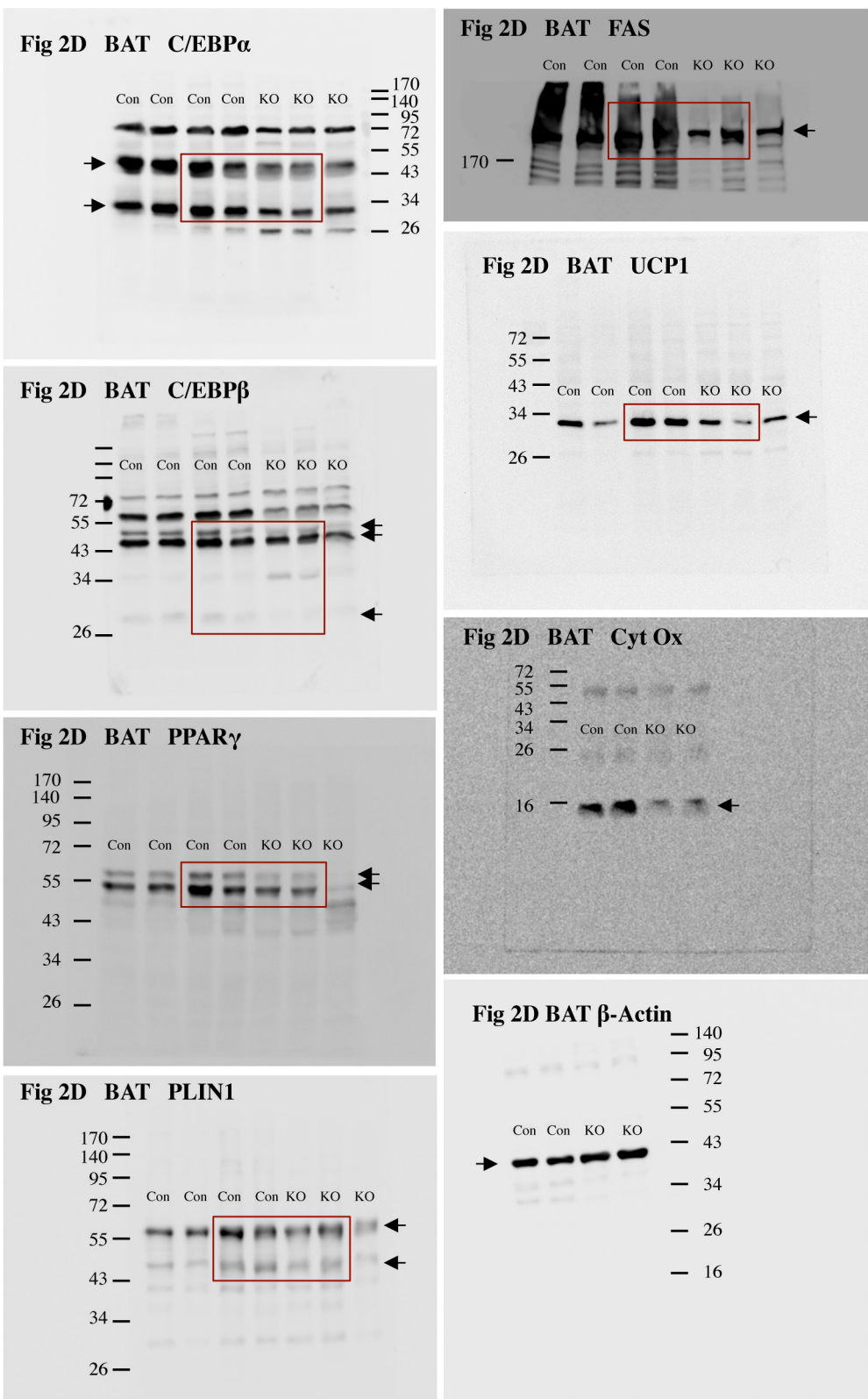

**Fig 2D**

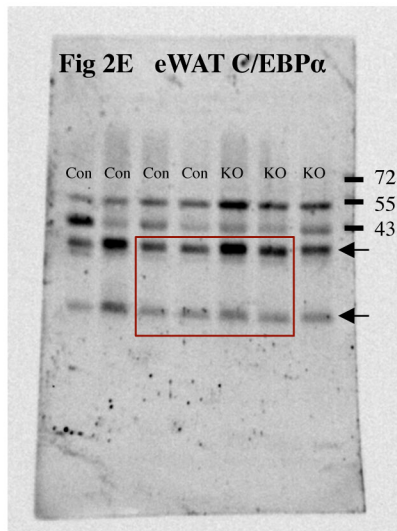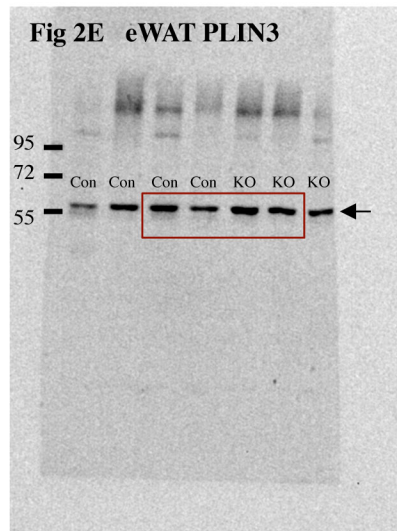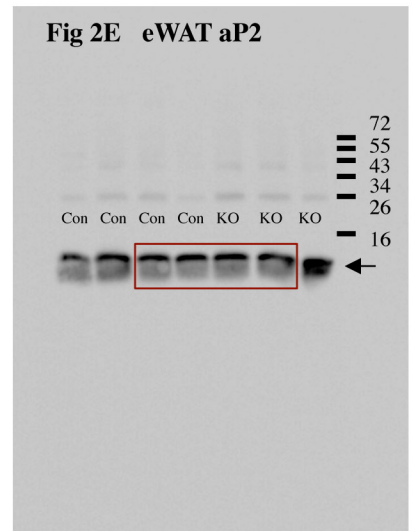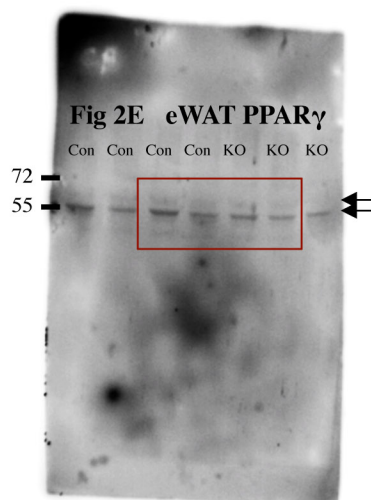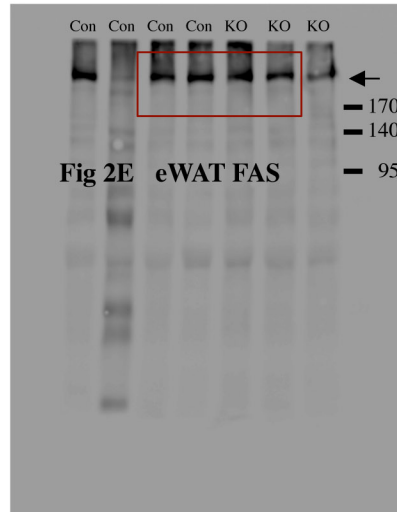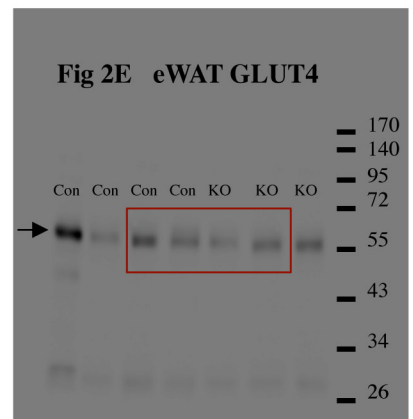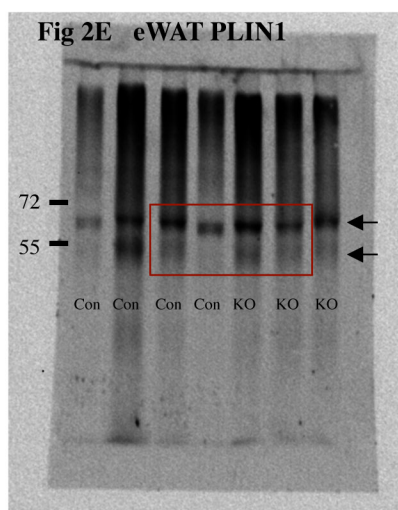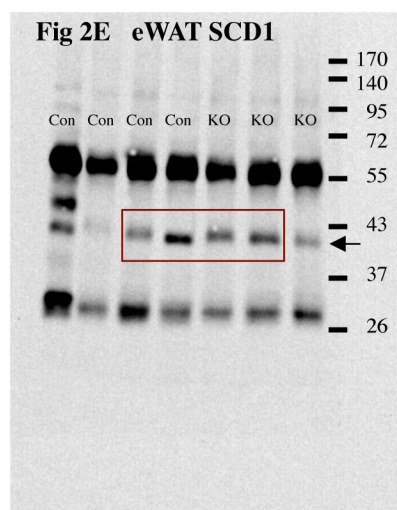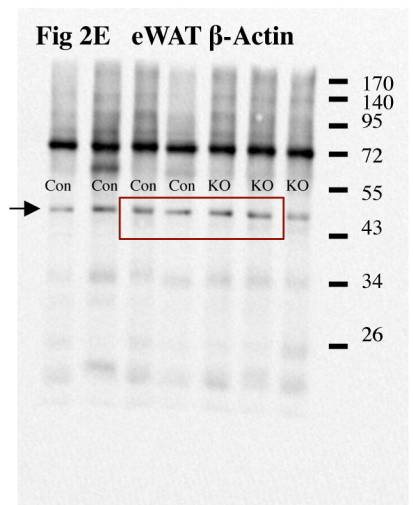

**Fig 2E**

Supplement: Source data for Figure 2 [file embor2013111df2.pdf]

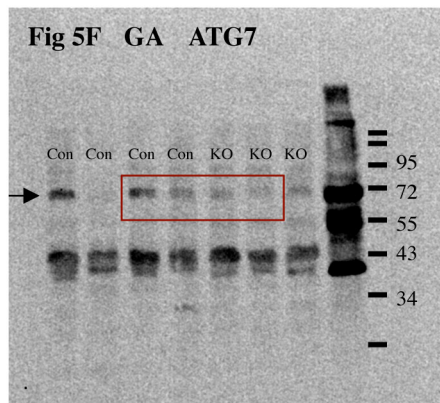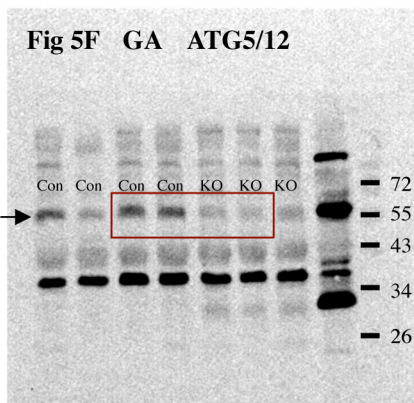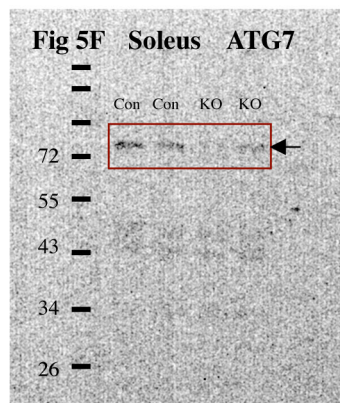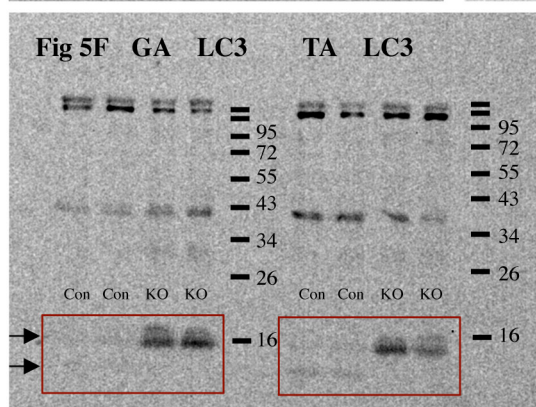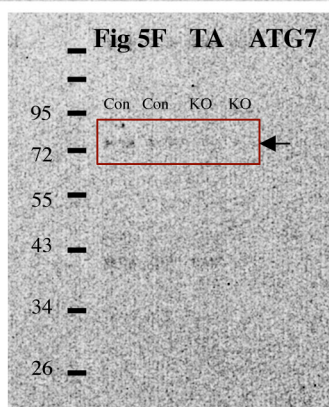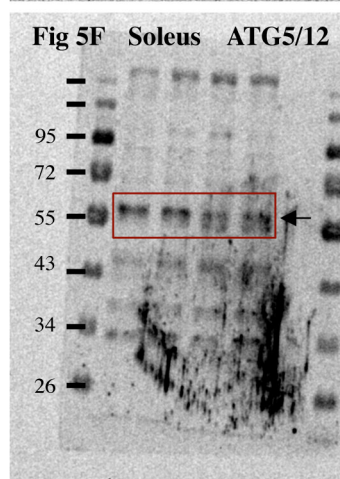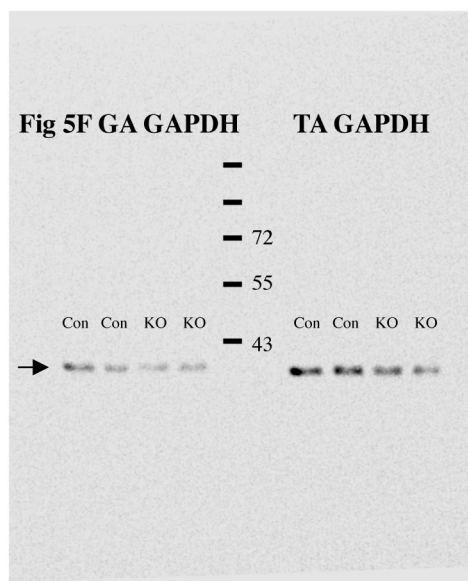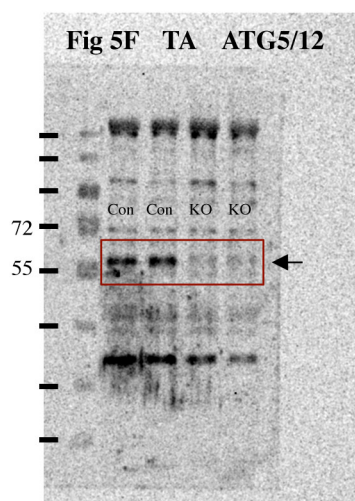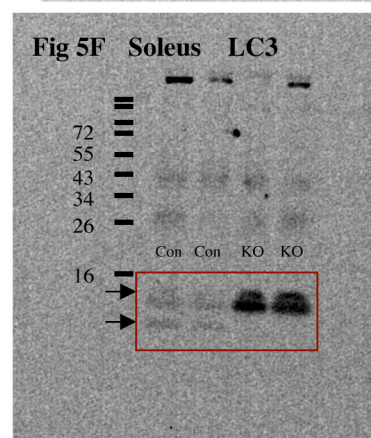

**Fig 5F**

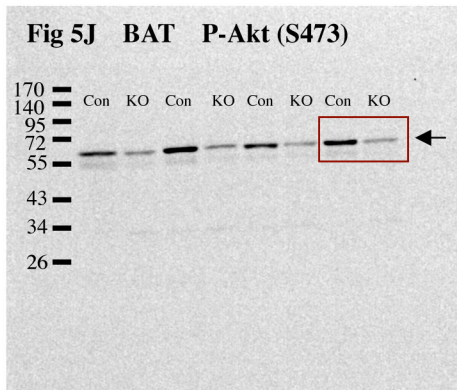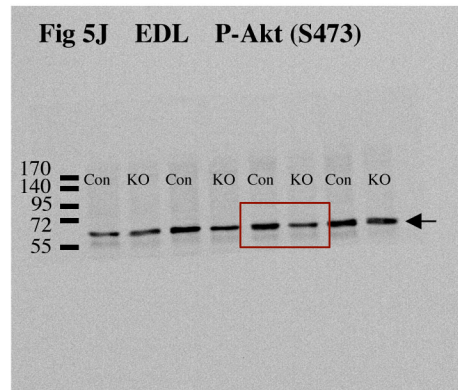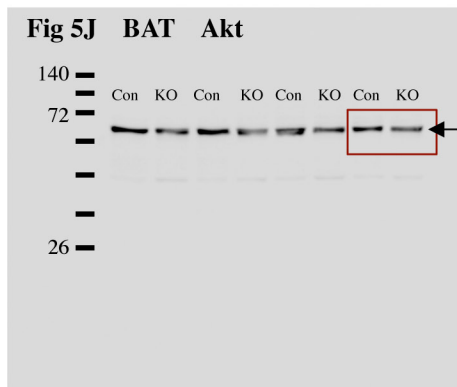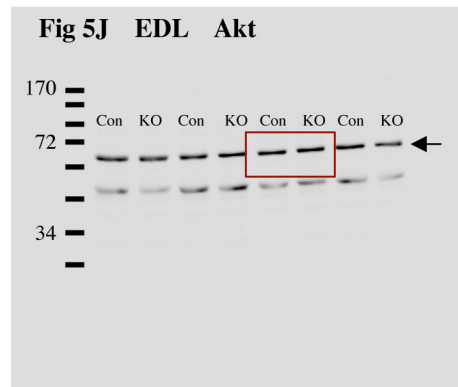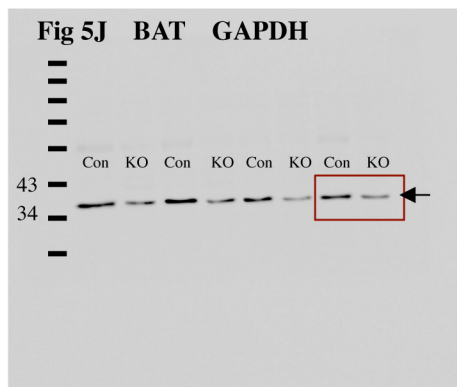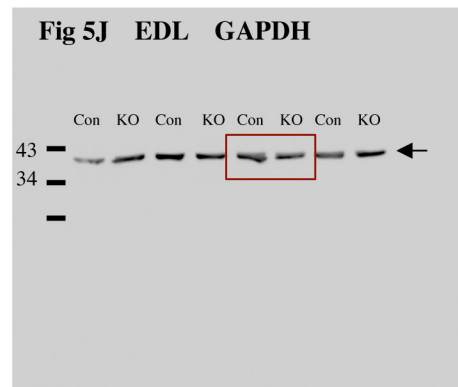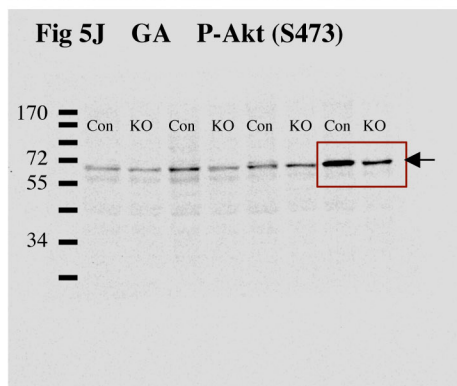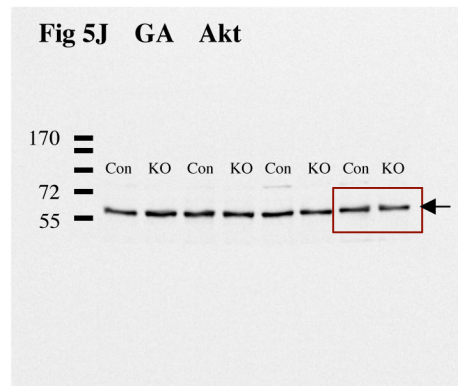

**Fig 5J**

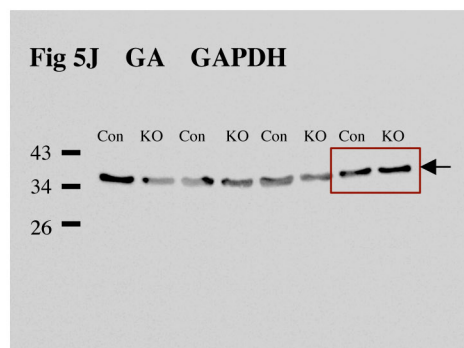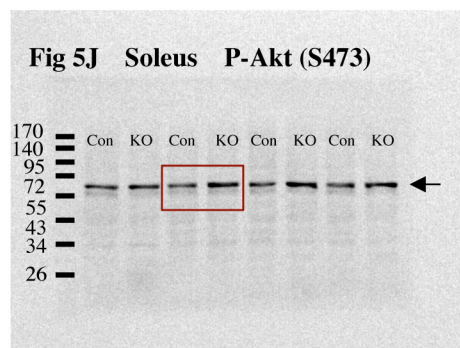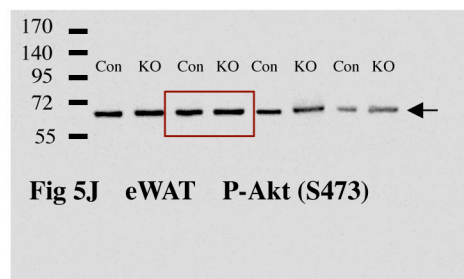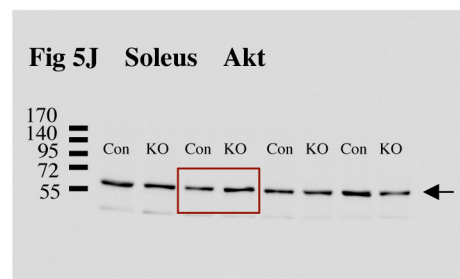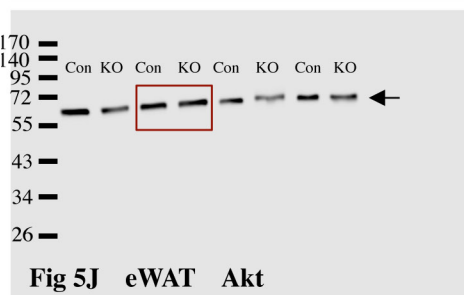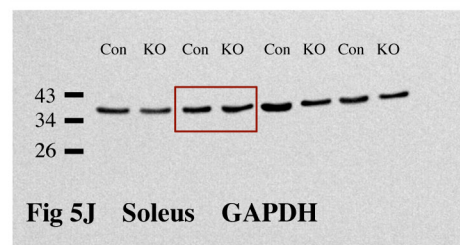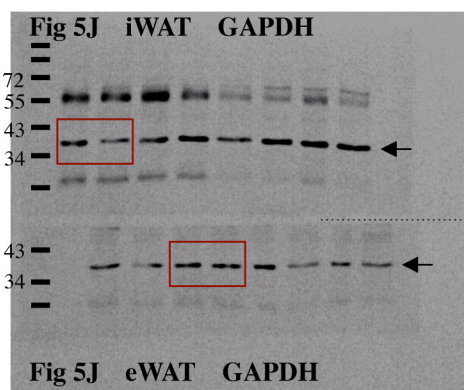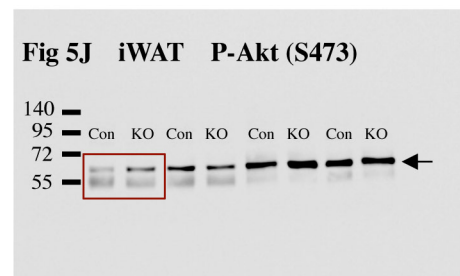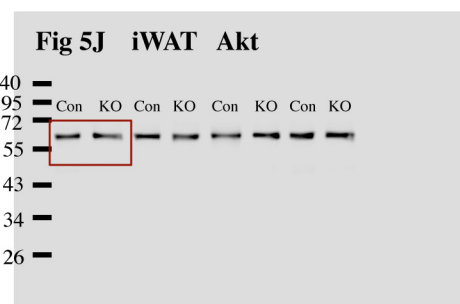

**Fig 5J**

Supplement: Source data for Figure 5 [file embor2013111df5.pdf]
