# Supplementary material for: Autophagy in Myf5+ progenitors regulates energy and glucose homeostasis through control of brown fat and skeletal muscle development
Source: EMBO Rep. 2013 Aug 2;14(9):795–803. doi: 10.1038/embor.2013.111 (PMC3790054; doi:10.1038/embor.2013.111)
Supplement: Review Process File [file embor2013111s2.pdf]

EMBO
reports

Manuscript EMBOR-2013-37564

Autophagy in Myf5+ progenitors regulates energy and glucose homeostasis through control of brown fat and skeletal muscle development

Nuria Martinez-Lopez, Diana Athonvarangkul, Srabani Sahu, Luisa Coletto, Haihong Zong, Claire C. Bastie, Jeffrey E. Pessin, Gary J. Schwartz and Rajat Singh

Corresponding author: Rajat Singh, Albert Einstein College of Medicine

Review timeline:

Submission date:	22 May 2013
Accepted:	28 June 2013

Editor: Nonia Pariente

Transaction Report:

Please note that the manuscript was previously reviewed at another journal and the reports were taken into account in the decision making process at EMBO reports. Since the original reviews are not subject to EMBO's transparent review process policy, the reports and author response cannot be published.

© European Molecular Biology Organization
